# AKT-mediated phosphorylation of ZDHHC5 promotes NOD1 palmitoylation and innate immune signaling

**DOI:** 10.3389/fimmu.2026.1819627

**Published:** 2026-06-09

**Authors:** Shaojie Mi, Yue Zhu, Qian Li, Xin Wang, Xuewu Guo, Yali Chen

**Affiliations:** 1 Key Laboratory of Industrial Fermentation Microbiology of Ministry of Education, Tianjin Industrial Microbiology Key Lab, College of Biotechnology of Tianjin University of Science and Technology, Tianjin, China; 2 State Key Laboratory of Medical Proteomics, National Center for Protein Sciences (Beijing), Academy of Military Medical Sciences, Beijing, China; 3 Cancer Center, Renmin Hospital of Wuhan University, Wuhan, Hubei, China

**Keywords:** AKT, growth factor signaling, NOD1 signaling, palmitoylation, phosphorylation, ZDHHC5

## Abstract

**Introduction:**

Nucleotide-binding oligomerization domain 1 (NOD1) is an intracellular pattern recognition receptor that detects bacterial peptidoglycan and initiates innate immune responses through membrane-associated signaling complexes. NOD1 activation depends on ZDHHC5-mediated palmitoylation, which promotes its membrane recruitment. However, whether growth factors and insulin modulate this NOD1 activation remains poorly defined.

**Methods:**

We investigated the effects of growth factors and insulin on NOD1 signaling using biochemical and cell-based approaches. Protein phosphorylation and interactions were analyzed by immunoblotting, co-immunoprecipitation, and mutagenesis assays. NOD1 palmitoylation, membrane localization, and downstream signaling activities were evaluated following modulation of AKT signaling and ZDHHC5 phosphorylation.

**Results:**

We found that growth factors and insulin positively regulate NOD1 activation through an AKT-ZDHHC5-NOD1 signaling axis. Mechanistically, AKT directly phosphorylated the palmitoyltransferase ZDHHC5 at Ser345 and Ser380, promoting its retention at the plasma membrane and enhancing its enzymatic activity toward NOD1. AKT-dependent phosphorylation increased NOD1 palmitoylation and membrane recruitment, thereby facilitating activation of downstream innate immune signaling pathways.

**Discussion:**

These findings identify a previously unrecognized mechanism linking growth factor- and insulin-mediated AKT activation to innate immune signaling. AKT-dependent phosphorylation of ZDHHC5 promotes NOD1 palmitoylation and activation, revealing a positive regulatory axis that integrates metabolic cues with innate immune responses. The AKT-ZDHHC5 pathway may therefore represent a potential target for modulating NOD1- driven inflammatory diseases.

## 1 Introduction

The innate immune system relies on pattern recognition receptors (PRRs) to detect pathogen-associated molecular patterns (PAMPs) and initiate host defense responses (1–3). Among these receptors, nucleotide-binding oligomerization domain-containing protein 1 (NOD1) is a cytosolic PRR that senses γ-D-glutamyl-meso-diaminopimelic acid (iE-DAP), a conserved peptidoglycan motif predominantly derived from Gram-negative bacteria (2, 4, 5).

Upon binding its ligand, NOD1 oligomerizes in an ATP-dependent manner and engages receptor-interacting serine/threonine-protein kinase 2 (RIPK2) through CARD–CARD interactions, which subsequently activates transforming growth factor β–activated kinase 1 (TAK1), the IκB kinase (IKK) complex, and downstream NF-κB and MAPK signaling pathways (6). This signaling cascade promotes the transcription of pro-inflammatory mediators, including *IL-6* and *CXCL-1/2 (*7). Aberrant NOD1 activation has been linked to a range of inflammatory and immune-related disorders, such as inflammatory bowel disease, uveitis, rheumatoid arthritis, and cancer, highlighting the importance of tight regulation of this pathway (8–10).

Growing evidence suggests that the subcellular distribution of NOD1 is a key factor in its signaling capacity. Effective activation of NOD1 depends on its recruitment to membrane-proximal compartments, where it can encounter bacterial peptidoglycan-derived ligands (3, 11). This membrane association is facilitated by S-palmitoylation, a reversible lipid modification catalyzed by the membrane-bound palmitoyl acyltransferase ZDHHC5 (12). Notably, disease-associated NOD1 variants often display impaired palmitoylation and defective membrane targeting, connecting abnormal lipid modification with dysregulated innate immune signaling. Although ZDHHC5-dependent palmitoylation is known to be critical for NOD1 activation, the upstream pathways that control ZDHHC5 activity remain poorly understood.

The serine/threonine kinase AKT is a central signaling node downstream of growth factors and cytokines, coordinating cell proliferation, survival, and metabolic adaptation across diverse cell types. Canonical growth factor signaling activates AKT through a well-established cascade: growth factor binding to receptor tyrosine kinases (RTKs) triggers phosphatidylinositol 3-kinase (PI3K) activation, which generates phosphatidylinositol (3–5)-trisphosphate (PIP3) at the plasma membrane. PIP3 recruits AKT via its pleckstrin homology (PH) domain, enabling phosphorylation at Thr308 by PDK1 and at Ser473 by mTORC2, resulting in full AKT activation (13, 14), allowing cells to integrate extracellular nutrient and mitogenic cues with intracellular anabolic programs. Beyond its classical role in growth control, AKT has also been implicated in innate immune signaling downstream of multiple PRRs, including Toll-like receptors (TLRs) and NOD-like receptors (NLRs), where it modulates inflammatory gene expression, cytokine production, and cell survival (15). However, whether AKT directly links growth factor–driven environmental cues to NOD1 activation, particularly through regulation of NOD1 spatial organization and membrane-proximal signaling, remains incompletely understood.

In this study, we identify growth factors and insulin could positively regulate NOD1 activation through AKT-mediated phosphorylation of ZDHHC5. We demonstrate that AKT phosphorylates ZDHHC5 at Ser345 and Ser380, enhancing its membrane association and enzymatic activity, thereby promoting NOD1 S-palmitoylation and efficient recruitment to membrane compartments. Disruption of AKT-dependent ZDHHC5 phosphorylation attenuates NOD1 palmitoylation and downstream inflammatory signaling. Collectively, our findings uncover a previously unappreciated mechanism by which growth factor–associated AKT signaling intersects with innate immunity and provide mechanistic insight into the regulation of NOD1 signaling in inflammatory diseases.

## 2 Materials and methods

### 2.1 Reagents

DMEM (11–965–118), FBS (10270-106) were purchased from Gibco; And the following reagents were used: polyethyleneimine (PEI) (23966-2) from Polysciences Inc.; Lipofectamine™ RNAiMAX (13778100), Prestained Protein Ladder (26616), Streptavidin Agarose (SA10004), EZ-Link BMCC-Biotin (21900)from Thermo Fisher; 2X MultiF Seamless Assembly Mix (RK21020) from ABclonal ABclonal Biotechnology; 2-Bromohexadecanoic (238422), N-Ethylmaleimide (E3876), hydroxylamine solution (467804), TBTA (Tris[(1-benzyl-1H-1, 2, 3-triazol-4-yl)methyl]amine (678937), Copper (II) sulfate pentahydrate (469130), TCEP (C4706), ANTI-FLAG^®^ M2 Affinity Gel (A2220), EZview™ Red Anti-HA Affinity Gel (E6779) from Millipore Sigma; C12-iE-DAP (tlrl-c12dap), from Invivogen; Biotin Picolyl Azide (1167), Alkynyl Palmitic Acid (1165) from Click Chemistry Tools; Mouse IL-6 (EMC004) ELISA kit from NeoBioscience; MK-2206 (HY-108232) from MCE.

### 2.2 Antibodies

The following antibodies were used in this study: NOD1 (3545), p38 MAPK (8690), Phospho-p38 MAPK (Thr180/Tyr182) (4511), Phospho-NF-kB p65 (Ser536) (3033), LDHA (3582), Phospho-Akt (Ser473) (D9E) Rabbit Monoclonal Antibody (4060), Akt Antibody (9272), Phospho-Akt Substrate (RXXS*/T*) (110B7E) Rabbit Monoclonal Antibody (9614), from CST; E-Cadherin (20874-1-AP), β-Actin (60008-1-Ig), from Proteintech; NOD1 (MABF215), ZDHHC5 (HPA014670), Monoclonal ANTI-FLAG M2 antibody (F1804), Anti-HA-tag (H9658), from Millipore Sigma; Biotin (ab53494), from Abcam; NF-kB p65 (sc-372), from Santa Cruz; Alexa Fluor^®^ 488 AffiniPure^®^ Goat Anti-Mouse IgG (H+L) (115-545-003), from Jackson ImmunoResearch Laboratories.

### 2.3 Cell culture

Immortalized BMDMs (iBMDMs) were kindly provided by Dr. Li Tao (16). HEK293T and iBMDM cells were cultured in DMEM supplemented with 10% fetal bovine serum (FBS) and 1% penicillin-streptomycin at 37 °C with 5% CO_2_. Cells were routinely screened for mycoplasma contamination by PCR and cultured under mycoplasma-free conditions.

Cells were transiently transfected with plasmids using PEI according to the manufacturer’s instructions. Briefly, cells were seeded in 6-well plates and transfected at approximately 70% confluency with 2 μg plasmid DNA per well. For cells cultured in 6-cm dishes, 6-8 μg plasmid DNA was used per dish. Transfection complexes were prepared in Opti-MEM and added to the cells for 6 h, after which the medium was replaced with complete DMEM. Cells were harvested 24–36 h post-transfection for subsequent analyses. For siRNA-mediated knockdown, cells were transfected with 50 nM siRNA using Lipofectamine RNAiMAX according to the manufacturer’s protocol. Cells were collected 48–72 h post-transfection for downstream assays.

For growth factor stimulation, HEK293T cells were serum-starved in DMEM without FBS for 12–16 h and then stimulated with either complete medium (10% FBS) or insulin (100 nM) for 30 min. iBMDMs were serum-starved in DMEM containing 0.5% FBS for 2 h, followed by continued starvation, serum refeeding (10% FBS), or insulin (100 nM) treatment in the presence of C12-iE-DAP (1 μg/mL) for 30 min. Cells were then harvested and subjected to immunoblotting and immunofluorescence analyses.

### 2.4 Plasmids

Full-length or truncated mutants of ZDHHC5 were inserted into FLAG-tagged (pCMV-3×FLAG), HA-tagged (pCMV-HA), GST-tagged (pGEX-4T-2), or pHAGE vectors to create lentiviral expression constructs. Similarly, full-length and truncated NOD1 sequences were cloned into the FLAG-tagged vector, while full-length AKT was cloned into the HA-tagged vector. N-myristoylation signal from Src kinase (17) was added to N-terminal of HA-AKT with PCR and cloned into pCMV-HA vector. All mutant constructs were generated using site-directed mutagenesis and verified by DNA sequencing. Full-length FLAG-NOD1 and GFP-NOD1 plasmids were generously provided by Dr. Dante Neculai.

RNA interference plasmids were constructed with pLKO.1. Lentiviral supernatants were produced by transiently transfecting 293T cells with the corresponding expression plasmids along with the helper plasmids pSPAX2 and pMD2G. Viral supernatants were collected 48 h after transfection, filtered through 0.45 μm membranes, and used to infect target cells in the presence of Polybrene (8 μg/ml).

The shRNA target sequences used in this study were as follows:

ZDHHC5-shRNA-1
Forward: 5′-cgcaatggaagcctatcttat-3′
Reverse: 5′-ataagataggcttccattgcg-3′
ZDHHC5-shRNA-2
Forward: 5′-ccaaagaaagagaagacaatt-3′
Reverse: 5′-aattgtcttctctttctttgg-3′

### 2.5 Immunoprecipitation

Cells transfected with the indicated plasmids were harvested and lysed on ice for 30 minutes in NETN buffer (10 mM Tris-HCl [pH 8.0], 100 mM NaCl, 1 mM EDTA, and 0.5% NP-40) supplemented with protease inhibitors, followed by a 15-second sonication. Lysates were cleared by centrifugation at 12, 000 g for 10 minutes at 4 °C. For immunoprecipitation, lysates were incubated with FLAG M2 or HA beads for 8 h at 4 °C with rotation. Beads were washed three times with NETN buffer, then boiled in 2× SDS loading buffer, and analyzed by immunoblotting using the indicated antibodies.

For endogenous immunoprecipitation (IP), cell lysates were incubated with indicated antibody for 6 h at 4 °C, followed by incubation with Protein A/G beads for 4 h at 4 °C. Beads were washed three times with NETN buffer, boiled in 2× SDS loading buffer, and subjected to immunoblot analysis with indicated antibodies.

### 2.6 *In vitro* kinase assay

GST-fused ZDHHC5 fragments (amino acids 254-404, encompassing S345 and S380) were generated in wild-type (WT) and mutant forms (S345A, S380A, and the double mutant S345A/S380A, hereafter referred to as 2A) and expressed in *E. coli* BL21 bacteria, followed by purification using standard glutathione affinity chromatography. Purified AKT was incubated with various substrates in kinase assay buffer (15 mM HEPES, pH 7.0, 450 μM dithiothreitol, 18.75 mM MgCl2, 6.25 mM β-glycerophosphate, 1.25mM EGTA and 125 μM ATP), with 150 μM AMP, at 30 °C for 30 min. Samples were analyzed by Western blot with indicated antibodies.

### 2.7 Immunofluorescent staining

Cells cultured on coverslips were fixed by 4% paraformaldehyde for 15 min at room temperature, followed by three washes with PBS. Cells were then permeabilized and blocked in PBS containing 0.1% saponin and 5% goat serum for 1 hour at room temperature. Samples were incubated with primary FLAG antibody diluted in PBS containing 0.05% saponin and 1% goat serum overnight at 4 °C. After three times PBS washes, cells were incubated with secondary antibody diluted in PBS containing 0.1% saponin and 5% goat serum for 1 h at room temperature. Fluorescent images were captured using a Nikon A1R confocal microscope and analyzed with Nikon NIS software.

### 2.8 Click chemistry reaction for palmitoylation

Click chemistry reaction for protein palmitoylation was detected as described (18). Briefly, cells were treated with 100 μM palmitate analogue alk-C16 for 8 h, then lysed in 200 μL of 1% SDS TEA buffer (150 mM NaCl, 50 mM triethanolamine, pH 7.4) supplemented with a protease inhibitor cocktail and 50 U/mL nuclease. Lysates were diluted with TEA buffer to achieve a final SDS concentration of 0.2%, and the target protein was immunopurified using anti-FLAG agarose beads. Bound proteins were eluted in 50 μL TEA buffer containing 4% SDS.

For click chemistry labeling, reagents were sequentially added: 0.5 μL of 10 mM biotin picolyl azide, 1 μL of 50 mM TCEP, 1 μL of 5 mM TBTA, and 1 μL of 50 mM CuSO_4_. The reaction mixture was thoroughly mixed and incubated at room temperature in the dark for 1 h. Samples were then mixed with 5× SDS loading buffer and heated at 95 °C for 10 min. Samples were analyzed by Western blotting with indicated antibodies.

### 2.9 Acyl-biotin exchange

Acyl–biotin exchange (ABE) assays were performed essentially as previously described (19). Briefly, samples were lysed in 150 μL lysis buffer (100 mM Tris-HCl pH 7.2, 150 mM NaCl, 2% SDS, with protease inhibitor cocktail) with 50 mM N-ethylmaleimide (NEM) and 50 U/ml nuclease. Lysates were centrifuged and supernatants were treated with 8 μL of 200 mM neutralized TCEP for 30 min at room temperature in the dark with rotation. Reduced thiols were subsequently blocked with NEM, and the reaction was stopped using methanol-chloroform-water precipitation. Samples were divided into two equal portions and incubated for 2 hours at room temperature in 150 μL lysis buffer containing either 0.75 M hydroxylamine or a negative control. Following another round of methanol-chloroform-water precipitation and brief air-drying, the samples were dissolved in 150 μL of lysis buffer containing 200 μM biotin-BMCC and gently mixed at room temperature for 2 h. The samples were precipitated again, dissolved in 120 μL lysis buffer, and aliquoted 20 μL was reserved as a loading control. The remaining 100 μL was diluted 1:10 with Tris buffer and incubated with 10 μL of streptavidin beads overnight at 4 °C with rotation. Beads were washed three times with PBS containing 1% SDS. Both the beads and loading control samples were mixed with SDS loading buffer and heated at 95 °C for 10 min. Proteins were resolved by SDS-PAGE and analyzed via Western blotting.

### 2.10 Subcellular fractionations assays

After the indicated stimulation, total cell lysates were prepared by lysing cells from a 10 cm dish in 1 mL of RIPA buffer on ice. Cytosolic and membrane fractions were separated using sucrose gradient fractionation as previously described (20). Briefly, cells were cultured in 10 cm dishes until reaching 90% confluency in a 37 °C incubator with 5% CO_2_. The cells were rinsed twice with ice-cold PBS and immediately resuspended in 1 mL of sucrose buffer (250 mM sucrose, 20 mM HEPES, 10 mM KCl, 1.5 mM MgCl_2_, 1 mM EDTA, 1 mM EGTA, 1 mM DTT, 1× protease inhibitor, and phosphatase inhibitor, pH 7.4) per dish on ice, followed by harvesting with a cell scraper.

The lysates were incubated on ice for 30 min and then sequentially centrifuged at 720 g for 5 min and 10, 000 g for 10 min at 4 °C, with supernatants transferred to fresh tubes after each step. To obtain the cytosolic fraction, the supernatant was ultracentrifuged at 100, 000 g for 1 h at 4 °C. The resulting cytosolic fraction was concentrated by methanol precipitation and resuspended in 2% SDS buffer (100 mM Tris-HCl, pH 7.2, 150 mM NaCl, 2% SDS, 1× protease inhibitor, and phosphatase inhibitor, pH 8.0). The pellet from ultracentrifugation, representing the membrane fraction, was washed in sucrose buffer and subjected to a second ultracentrifugation at 100, 000 × g for 1 h at 4 °C, then resuspended in 2% SDS buffer. Both fractions were resolved by SDS-PAGE and analyzed via Western blotting.

### 2.11 ELISA

iBMDMs were seeded in 6-well plates and serum-starved in DMEM containing 0.5% FBS for 2 h. Cells were then either maintained under serum starvation conditions, stimulated with complete medium (10% FBS), or treated with insulin (100 nM), in the presence of C12-iE-DAP (5 μg/mL) for 6 h. Culture supernatants were collected, and IL-6 levels were measured using a mouse IL-6 ELISA kit according to the manufacturer’s instructions.

### 2.12 Data analysis

Band intensities in Western blots were quantified using ImageJ software. Protein levels were normalized to the corresponding loading control (e.g., E-cadherin). Quantification was performed from at least three independent experiments. For quantification of plasma membrane-associated protein fluorescence intensity, individual cells were manually outlined, and a membrane-associated region of interest (ROI) was generated by expanding the selection by 3 pixels. Mean fluorescence intensity within the membrane ROI was quantified on a per-cell basis using ImageJ. The ROI width was defined consistently across all samples (12, 21).At least 30 cells per condition were analyzed. Statistical analyses were conducted in GraphPad Prism, based on at least three independent biological replicates. Depending on the experimental design, unpaired t-tests, one-way ANOVA, and two-way ANOVA were used as appropriate. Data were presented as mean ± SEM, and p < 0.05 was considered statistically significant.

## 3 Results

### 3.1 Cellular growth conditions regulate NOD1 signaling

**Figure 1:**
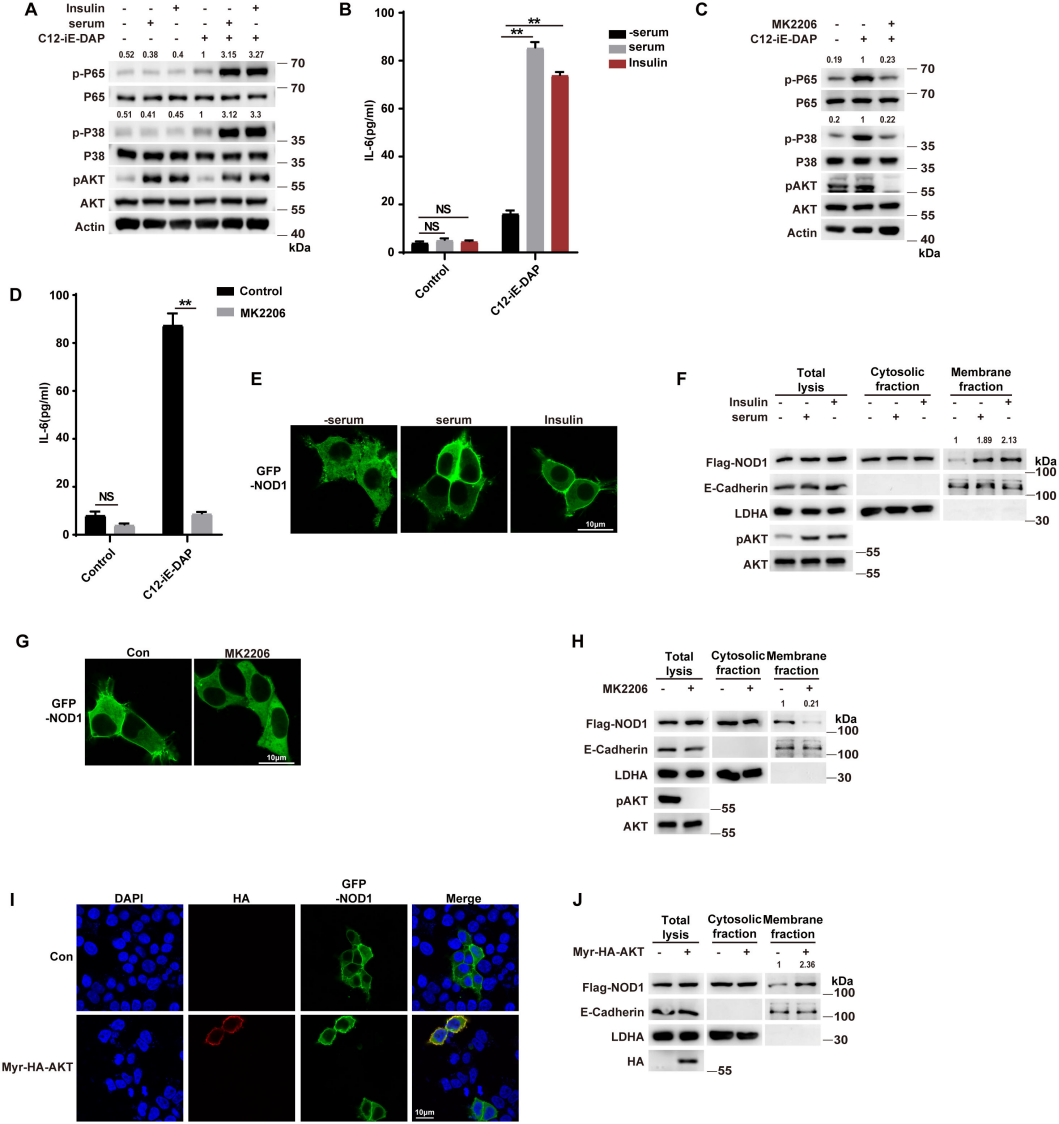
Cellular growth conditions regulate NOD1 signaling. **(A)** Mouse iBMDMs were serum-starved for 2 h, followed by continued serum starvation, serum refeeding, or insulin treatment in parallel with C12-iE-DAP (1 μg/ml) stimulation for 30 min, and p65 and p38 phosphorylation were analyzed by immunoblotting. **(B)** Mouse iBMDMs were serum-starved for 2 h, followed by 0.5% serum, serum refeeding, or insulin treatment in parallel with C12-iE-DAP (5 μg/ml) for 7 h. The IL-6 release in the medium was measured with ELISA. For each experimental group, three supernatant samples from 3 replicated cell culture were analyzed. **p < 0.01, NS, p > 0.05. mean ± SEM, two-way ANOVA followed by Sidak’s multiple comparisons test. **(C)** Mouse iBMDM cells were treated with MK2206 (2 μM) in serum-free medium for 1 h, then stimulated with C12-iE-DAP (1 μg/ml) for 30 min, p65 and p38 phosphorylation were analyzed by immunoblotting. **(D)** Mouse iBMDM cells were treated with MK2206 (0.5 μM) in serum-free medium and stimulated with C12-iE-DAP (5 μg/ml) for 7 h. The IL-6 release in the medium was measured with ELISA. For each experimental group, three supernatant samples were analyzed. **p < 0.01, NS, p > 0.05. mean ± SEM, two-way ANOVA followed by Sidak’s multiple comparisons test. **(E)** HEK293T were serum-starved for 16 h, followed by continued serum starvation, serum refeeding, or insulin treatment. Representative fluorescence images showing the localization of GFP-NOD1 were presented. **(F)** HEK293T were serum-starved for 16 h, followed by continued serum starvation, serum refeeding, or insulin treatment. Total, cytosolic, and membrane fractions were immunoblotted with indicated antibodies. **(G)** HEK293T were treated with MK2206 (2 μM) in serum-free medium for 1 h. Representative fluorescence images showing the localization of GFP-NOD1 were presented. **(H)** HEK-293T cells expressing FLAG-NOD1 were treated with MK2206 (2 μM) in serum-free medium for 1 h. Total, cytosolic, and membrane fractions were immunoblotted with indicated antibodies. **(I)** HEK293T cells were transfected with the indicated plasmids. Representative fluorescence images showing the localization of GFP-NOD1 (green) and MYR-HA-AKT (red) are presented. **(J)** HEK293T cells were transfected with indicated plasmids. Total, cytosolic, and membrane fractions were immunoblotted with indicated antibodies.

Innate immune activation is tightly coupled to the cellular metabolic and growth environment (22–24). To examine how changes in extracellular nutrient and growth factor availability influence NOD1 responsiveness, immortalized bone marrow–derived macrophages (iBMDMs) were subjected to serum starvation followed by continued starvation, serum refeeding, or insulin supplementation and then stimulated with the NOD1 agonist C12-iE-DAP. Under serum refeeding and insulin treatment conditions, NOD1 stimulation induced robust phosphorylation of p65 and p38, indicative of activation of the NOD1 downstream NF-κB and MAPK pathways (**Figure 1A**). In contrast, serum-limited conditions were associated with a markedly attenuated NF-κB and MAPK response.

Consistent with these signaling changes, cytokine production was markedly influenced by the cellular growth state. iBMDMs maintained under serum refeeding or insulin-supplemented conditions produced significantly higher levels of IL-6 following prolonged C12-iE-DAP stimulation compared with serum-starved cells (**Figure 1B**). These findings suggest that NOD1-mediated innate immune signaling is tightly regulated by the surrounding growth factor environment, with cellular conditions that support efficient kinase activation and downstream inflammatory responses.

### 3.2 AKT activation enhances NOD1 signaling and membrane association

Given that growth factor availability robustly engages the AKT pathway (25–27), we next assessed whether AKT activity underlies the modulation of NOD1 signaling observed under these conditions. Pharmacological inhibition of AKT with MK2206 in iBMDMs markedly attenuated C12-iE-DAP–induced phosphorylation of p65 and p38 and significantly reduced IL-6 production (**Figures 1C**; **Supplementary Figures S1A, D**), demonstrating that AKT signaling contributes to NOD1-dependent signaling. Consistantly, knocking down AKT with siRNA significantly attenuated the phosphorylation of NF-kB p65 and MAPK p38, indicating that AKT plays a critical role in NOD1 signaling (**Supplementary Figure S1B****).**

Proper activation of NOD1 relies on its recruitment to the membrane. In HEK293T cells expressing GFP-NOD1, serum refeeding or insulin treatment resulted in a marked accumulation of NOD1 at the cell surface, whereas AKT inhibition caused a redistribution toward the cytosolic compartment, as revealed by fluorescence imaging (**Figures 1E**; **Supplementary Figures S1C, G, E**). These localization changes were further supported by biochemical fractionation, which showed a corresponding increase in membrane-associated NOD1 under AKT-activating conditions and a marked reduction following MK2206 treatment (**Figures 1F**; **Supplementary Figures S1D, H**).

Myristoylated AKT (Myr-AKT) is a constitutively active form of the AKT kinase engineered by adding a myristoylation signal sequence. Myr-AKT anchors to the membrane permanently, leading to constitutive phosphorylation of AKT and signaling activity (28, 29). Modulation of AKT signaling through expression of MYR-HA-AKT further altered the subcellular distribution of NOD1, strengthening the direct association between AKT activity and NOD1 membrane targeting (**Figures 1I**; **Supplementary Figures S1F, J**). These data support a role for AKT in linking growth factor–dependent cellular states to NOD1 membrane organization and downstream innate immune signaling.

### 3.3 AKT activation promotes NOD1 palmitoylation

**Figure 2:**
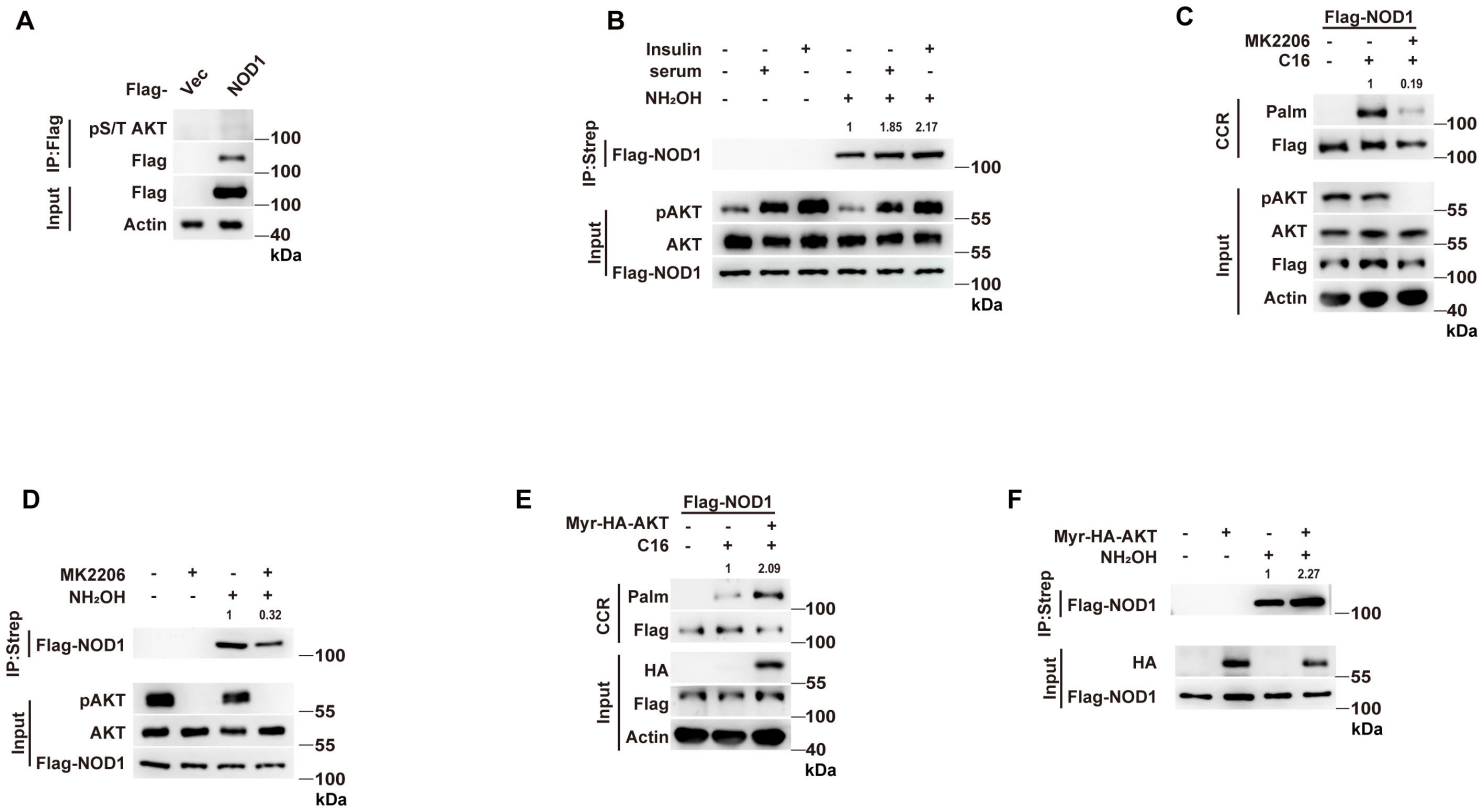
AKT activation enhances NOD1 palmitoylation. **(A)** HEK293T cells were transfected with FLAG-NOD1, and immunoprecipitation was performed using FLAG M2 beads. The immunoprecipitates were then analyzed by Western blotting with a Phospho-AKT Substrate motif antibody. **(B)** HEK293T cells expressing FLAG-NOD1 were serum-starved for 16 h, followed by continued serum starvation, serum refeeding, or insulin treatment. The level of NOD1 palmitoylation was detected by the ABE method. **(C)** HEK293T cells expressing FLAG-NOD1 were treated with MK2206 (1 μM) and labelled with alkyne-palmitic acid (alk-C16) for 8 h. The level of NOD1 palmitoylation was detected by click chemistry reaction. **(D)** HEK293T cells expressing FLAG-NOD1 were treated with MK2206 (2 μM) in serum-free medium for 1 h. The level of NOD1 palmitoylation was detected by the ABE method. **(E)** HEK293T cells were transfected with indicated plasmids and labelled with alkyne-palmitic acid (alk-C16) for 8 h. The level of NOD1 palmitoylation was detected by click chemistry reaction. **(F)** HEK293T cells were transfected with indicated plasmids. The level of NOD1 palmitoylation was detected by the ABE method.

Considering the critical role of post-translational modifications in NOD1 membrane association and signaling competence (12, 30, 31), we next examined whether AKT directly phosphorylates NOD1 as a potential regulatory mechanism. In HEK293T cells expressing FLAG-tagged NOD1, immunoprecipitation followed by immunoblotting with a phospho-AKT substrate motif antibody failed to detect a discernible AKT-dependent phosphorylation signal on NOD1 (**Figure 2A**), indicating that NOD1 is unlikely to be a direct substrate of AKT.

Since NOD1 palmitoylation is essential for its membrane localization, we tested whether AKT affects this modification. Cells were subjected to serum starvation followed by continued starvation, serum refeeding, or insulin treatment. Using the acyl-biotin exchange (ABE) assay, we observed that conditions associated with AKT activation, including serum refeeding and insulin stimulation, significantly increased the palmitoylation level of NOD1 compared with serum-limited conditions (**Figures 2B**; **Supplementary Figure S2A**). Conversely, pharmacological inhibition of AKT with MK2206 markedly reduced NOD1 palmitoylation, as detected by both metabolic labeling with alkyne-palmitic acid followed by click chemistry (**Figure 2C**) and ABE analysis (**Figure 2D**), demonstrating that AKT activity is required to maintain this modification.

Then HEK293T cells were transfected with MYR-HA-AKT to hyperactivate the AKT signaling and the NOD1 palmitoylation was analyzed. Ectopic expression of MYR-HA-AKT led to marked increase in NOD1 palmitoylation as detected by click chemistry–based labeling (**Figure 2E**) and was independently confirmed by ABE analysis (**Figure 2F**). These results demonstrate a positive regulatory link between AKT activity and NOD1 palmitoylation. This AKT-dependent increase in NOD1 palmitoylation provides a mechanistic explanation for its enhanced membrane targeting and downstream innate immune signaling under AKT-activated cellular states.

### 3.4 AKT interacts with and phosphorylates ZDHHC5

**Figure 3:**
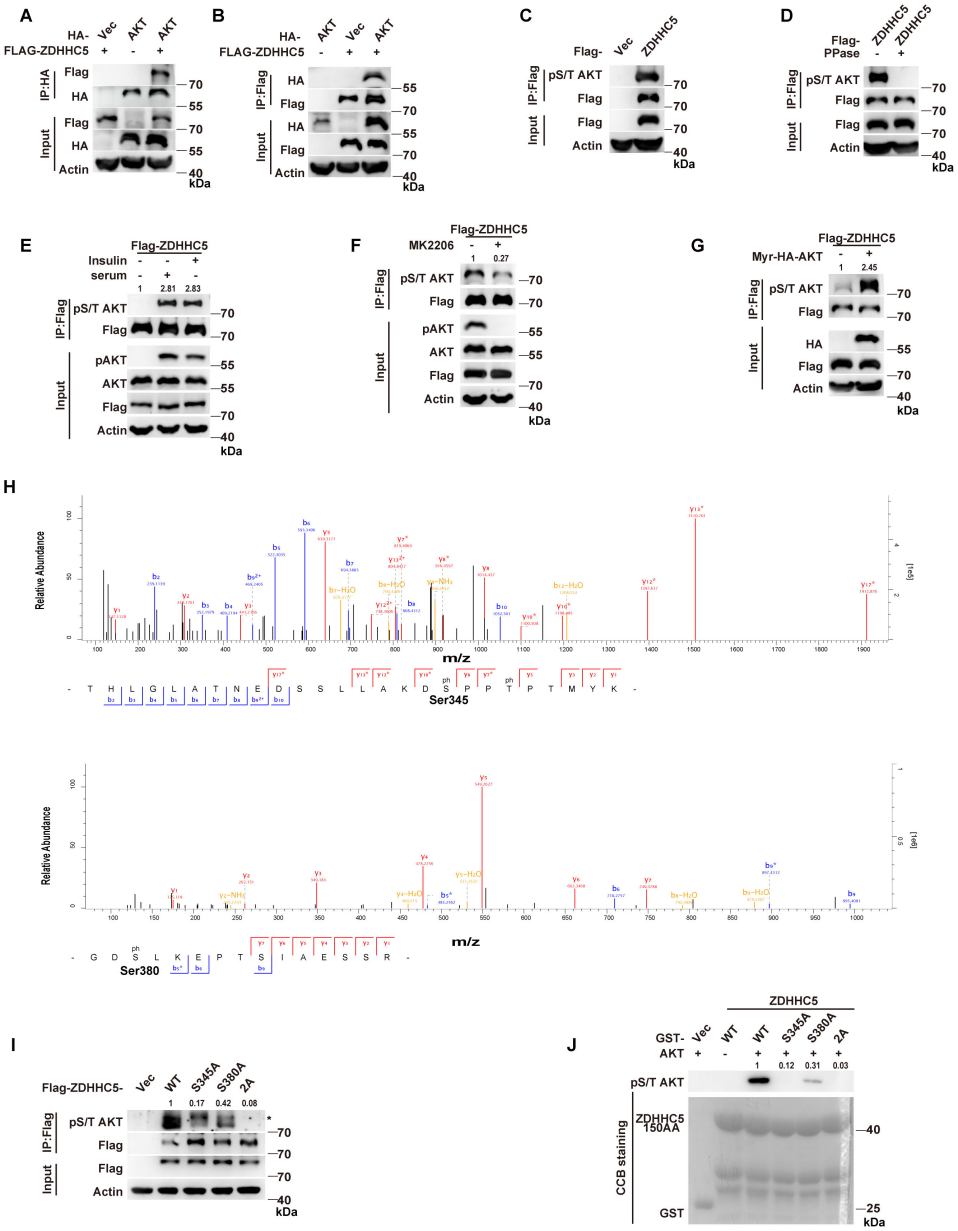
AKT interacts with and phosphorylates ZDHHC5. **(A)** HEK293T cells were transfected with FLAG-ZDHHC5 and HA-AKT. The interaction between ZDHHC5 and AKT were investigated by immunoprecipitation and immunoblot with indicated antibodies. IP with anti-HA antibody and blot with anti-FLAG or HA antibody, respectively. **(B)** HEK293T cells were transfected with FLAG-ZDHHC5 and HA-AKT. The interaction between ZDHHC5 and AKT were investigated by immunoprecipitation and immunoblot with indicated antibodies. IP with anti-FLAG antibody and blot with anti-FLAG or HA antibody, respectively. **(C)** HEK293T cells were transfected with FLAG-ZDHHC5, and immunoprecipitation was performed using FLAG M2 beads. The immunoprecipitates were then analyzed by Western blotting with a Phospho-AKT Substrate motif antibody. **(D)** HEK293T cells were transfected with FLAG-ZDHHC5, and immunoprecipitation was performed using FLAG M2 beads. The immunoprecipitates were treated with λ-phosphatase (λppase) at 30 °C for 30 min and analyzed by Western blotting with n Phospho-AKT Substrate motif antibody. **(E)** HEK293T cells were transfected with FLAG-ZDHHC5 were serum-starved for 16 h, followed by continued serum starvation, serum refeeding, or insulin treatment. Immunoprecipitation was then performed using FLAG M2 beads, and the immunoprecipitates were analyzed by Western blotting with a Phospho-AKT Substrate motif antibody. **(F)** HEK293T cells were transfected with FLAG-ZDHHC5 treated with MK2206 (2 μM) in serum-free medium for 1 h. Immunoprecipitation was then performed using FLAG M2 beads, and the immunoprecipitates were analyzed by Western blotting with a Phospho-AKT Substrate motif antibody. **(G)** HEK293T cells were transfected with indicated plasmids. Immunoprecipitation was then performed using FLAG M2 beads, and the immunoprecipitates were analyzed by Western blotting with a Phospho-AKT Substrate motif antibody. **(H)** Identification of potential phosphorylation sites on ZDHHC5 by mass spectrometry. The MS/MS spectra for phosphosites S345 (top) and S380 (bottom) were shown. **(I)** HEK293T cells were transfected with indicated plasmids, and immunoprecipitation was performed using FLAG M2 beads. The immunoprecipitates were then analyzed by Western blotting with a Phospho-AKT Substrate motif antibody. Asterisk, non-specific band., **(J)** *in vitro* kinase assay for ZDHHC5. Indicated GST protein was incubated with AKT in kinase buffer supplemented with 125 μM ATP and 150 μM AMP for 30 min at 30 °C, and then analyzed with Phospho-AKT Substrate motif antibody.

As the palmitoyl acyltransferase mediating NOD1 palmitoylation, ZDHHC5 represents a likely downstream target of AKT signaling, especially since AKT-dependent phosphorylation of NOD1 was not detected. Co-immunoprecipitation assays in HEK293T cells co-expressing FLAG-ZDHHC5 and HA-AKT revealed a robust physical interaction between the two proteins. Both anti-HA (**Figure 3A**) and anti-FLAG (**Figure 3B**) immunoprecipitation approaches detected ZDHHC5 together with AKT, consistent with an interaction between the two proteins in cells.

FLAG-tagged ZDHHC5 was immunoprecipitated and analyzed using an antibody recognizing the phosphorylated AKT substrate consensus motif to examine its phosphorylation status. A strong phosphorylation signal was detected on ZDHHC5 (**Figure 3C**). This signal was abolished after λ-phosphatase treatment, indicating that the signal represents specific phosphorylation rather than nonspecific antibody binding (**Figure 3D**).

We next assessed whether this modification is regulated by physiological AKT activity. Serum refeeding or insulin stimulation markedly increased the AKT substrate motif signal on ZDHHC5 compared with continued serum starvation (**Figure 3E**; **Supplementary Figure S3A**). In contrast, pharmacological inhibition of AKT with MK2206 substantially decreased ZDHHC5 phosphorylation (**Figure 3F**), indicating that AKT activity is required to maintain this modification. Similarly, expression of MYR-HA-AKT further enhanced ZDHHC5 phosphorylation, supporting a direct link between AKT signaling and ZDHHC5 phosphorylation (**Figure 3G**).

To identify potential AKT-mediated phosphorylation sites of ZDHHC5, FLAG-ZDHHC5 was affinity-purified from HEK293T cells co-expressing MYR-HA-AKT and subjected to mass spectrometry analysis. Three phosphopeptides derived from ZDHHC5 were identified with high-confidence phosphorylation sites (Phospho (STY) probability > 0.98), corresponding to Ser345, Ser380 and Ser621 (**Figure 3H**; **Supplementary Figure 3B**). Inspection of the MS/MS spectra revealed well-resolved fragment ion series supporting phosphorylation at these residues, as indicated by the diagnostic mass shifts on the corresponding b/y ions. Sequence analysis revealed that the region surrounding Ser345 (LLAKDS*PPT) contains a basic residue at the -3 position relative to the phosphoserine, partially matching the canonical AKT consensus motif (RxRxxS/T), whereas the sequences surrounding S380 and S621 display weaker conformity to this motif (32).

To determine the functional relevance of these sites, site-directed mutagenesis was performed. Substitution of Ser345 with alanine (S345A) resulted in a marked reduction in the AKT substrate motif signal, while mutation of Ser380 (S380A) caused a moderate decrease. In contrast, mutation of Ser621 (S621A) had no detectable effect (**Figure 3I**; **Supplementary Figure S3C**). Importantly, simultaneous mutation of Ser345 and Ser380 (2A) completely abolished the AKT substrate motif signal. Collectively, these results link the MS-identified phosphorylation sites to functional AKT-mediated modification of ZDHHC5, and demonstrate that Ser345 and Ser380 are AKT-responsive residues, with Ser345 likely serving as the predominant phosphorylation site.

Notably, an *in vitro* kinase assay confirmed that AKT directly phosphorylates ZDHHC5. Incubation of GST-tagged ZDHHC5 with recombinant AKT in the presence of ATP resulted in a strong phospho-AKT substrate motif signal, whereas mutation of the identified serine residues markedly impaired this phosphorylation (**Figure 3J**). Collectively, these data establish ZDHHC5 as a direct substrate of AKT and provide a mechanistic link between AKT activation and the regulation of NOD1 palmitoylation via AKT-dependent phosphorylation of ZDHHC5.

### 3.5 AKT-dependent phosphorylation facilitates ZDHHC5 membrane association

**Figure 4:**
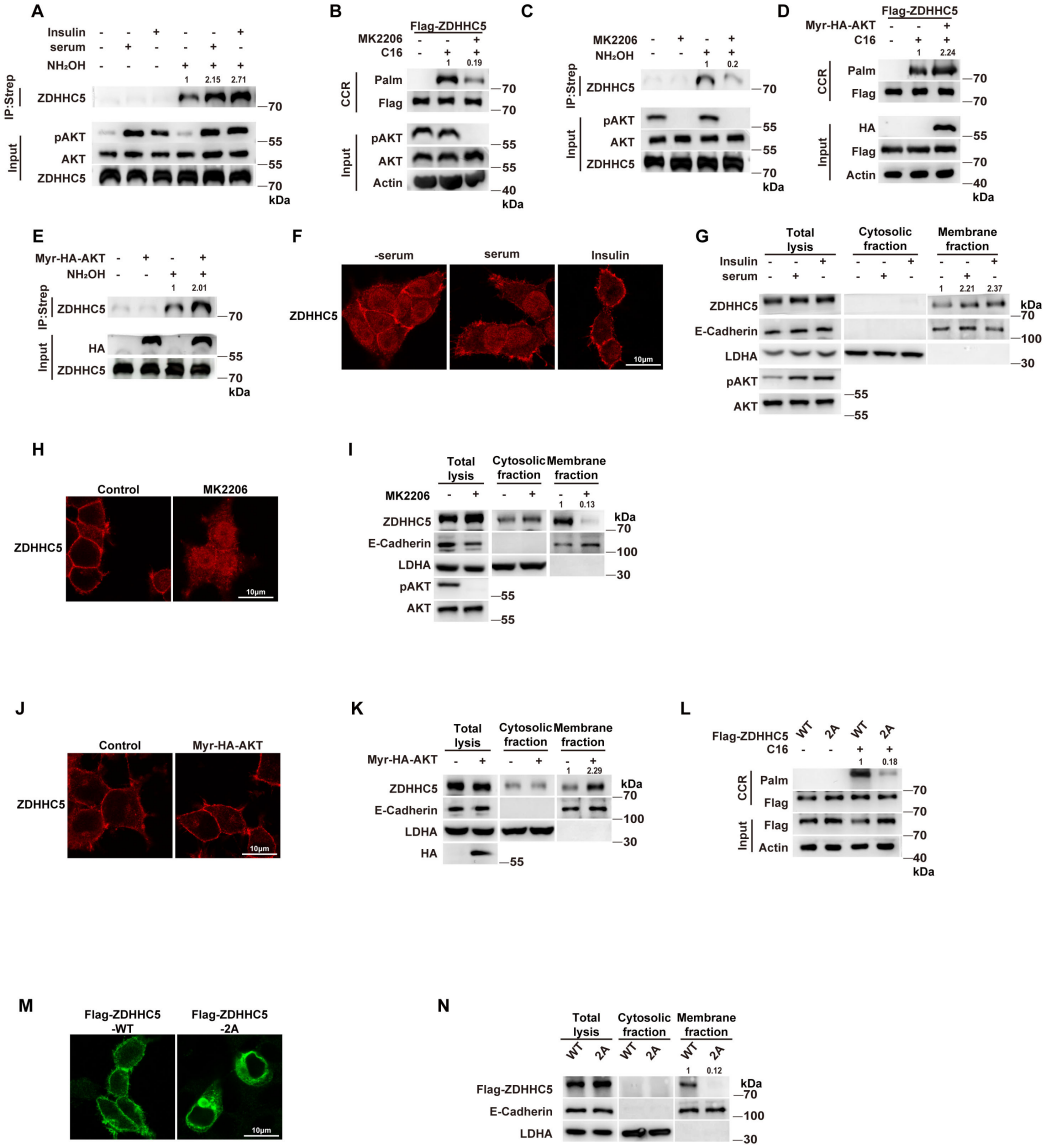
AKT-dependent phosphorylation facilitates ZDHHC5 membrane association. **(A)** HEK293T cells were serum-starved for 16 h, followed by continued serum starvation, serum refeeding, or insulin treatment. The level of ZDHHC5 palmitoylation was detected by the ABE method. **(B)** HEK293T cells expressing FLAG-ZDHHC5 were treated with MK2206 (1 μM) and labelled with alkyne-palmitic acid (alk-C16) for 8 h. The level of ZDHHC5 palmitoylation was detected by click chemistry reaction. **(C)** HEK293T cells were treated with MK2206 (2 μM) in serum-free medium for 1 h. The level of ZDHHC5 palmitoylation was detected by the ABE method. **(D)** HEK293T cells were transfected with indicated plasmids and labelled with alkyne-palmitic acid (alk-C16) for 8 h. The level of ZDHHC5 palmitoylation was detected by click chemistry reaction. **(E)** HEK293T cells were transfected with indicated plasmids. The level of ZDHHC5 palmitoylation was detected by the ABE method. **(F)** HEK293T cells were serum-starved for 16 h, followed by continued serum starvation, serum refeeding, or insulin treatment. Representative fluorescence images showing the localization of endogenous ZDHHC5 were presented. **(G)** HEK293T cells were serum-starved for 16 h, followed by continued serum starvation, serum refeeding, or insulin treatment. Total, cytosolic and membrane fractions of endogenous ZDHHC5 were immunoblotted with indicated antibodies. **(H)** HEK293T cells were treated with MK2206 (2 μM) in serum-free medium for 1 h. Representative fluorescence images showing the localization of endogenous ZDHHC5 were presented. **(I)** HEK293T cells were treated with MK2206 (2 μM) in serum-free medium for 1 h. Total, cytosolic and membrane fractions of endogenous ZDHHC5 were immunoblotted with indicated antibodies. **(J)** HEK293T cells were transfected with indicated plasmids. Representative fluorescence images showing the localization of endogenous ZDHHC5 were presented. **(K)** HEK293T cells were transfected with indicated plasmids. Total, cytosolic and membrane fractions of endogenous ZDHHC5 were immunoblotted with indicated antibodies. **(L)** HEK293T cells were transfected with indicated plasmids and labelled with alkyne-palmitic acid (alk-C16) for 8 h. The level of ZDHHC5 palmitoylation was detected by click chemistry reaction. **(M)** HEK293T cells were transfected with indicated plasmids. Representative fluorescence images showing the localization of FLAG-ZDHHC5-WT or 2A were presented. **(N)**HEK-293T cells expressing FLAG-ZDHHC5-WT or 2A. Total, cytosolic, and membrane fractions were immunoblotted with indicated antibodies.

ZDHHC5 activity is closely linked to its own palmitoylation, which supports its recruitment to the membrane (33, 34). Based on this, we hypothesized that AKT-dependent phosphorylation modulates the palmitoylation and membrane association of ZDHHC5. In HEK293T cells subjected to serum starvation followed by continued starvation, serum refeeding, or insulin stimulation, acyl-biotin exchange (ABE) analysis revealed a marked increase in ZDHHC5 palmitoylation under conditions associated with AKT activation (**Figure 4A**). In contrast, pharmacological inhibition of AKT with MK2206 significantly reduced ZDHHC5 palmitoylation, as detected by both click chemistry (**Figure 4B**) and ABE assays (**Figure 4C**).

HEK293T cells were transfected with MYR-HA-AKT and subjected to palmitoylation assays to examine the link between AKT signaling strength and ZDHHC5 palmitoylation. Enhanced AKT activity resulted in elevated levels of ZDHHC5 palmitoylation, whereas attenuation of AKT signaling led to a pronounced reduction in this modification (**Figures 4D, E**). Consistently, click chemistry assay further confirmed that modulation of AKT signaling directly correlates with the level of ZDHHC5 palmitoylation.

Consequently, we investigated whether AKT activity directly regulates the subcellular localization of ZDHHC5. Immunofluorescence imaging of endogenous ZDHHC5 revealed increased membrane localization following serum refeeding or insulin stimulation, whereas MK2206-induced AKT inhibition reduced the membrane localization of ZDHHC5 (**Figure 4F**; **Supplementary Figures S4A, B, H, C, D****).** These findings were further supported by biochemical fractionation, which showed a corresponding increase or decrease in the membrane-associated ZDHHC5 under AKT-activated or AKT-inhibited conditions, respectively (**Figure 4G**; **Supplementary Figures S4E, I**). Furthermore, overexpression of MYR-HA-AKT enhanced the membrane localization of ZDHHC5, strengthening the association between AKT activity and ZDHHC5 membrane association (**Figure 4J**; **Supplementary Figures S4F, K**).

Analysis of a phosphorylation-deficient ZDHHC5 mutant (2A) demonstrated significantly reduced palmitoylation and impaired membrane localization compared to the wild-type protein (**Figures 4L, M**; **Supplementary Figures S4G, N**). These results indicate that AKT-dependent phosphorylation enhances ZDHHC5 palmitoylation and promotes its membrane association, offering a mechanistic basis for the regulation of downstream NOD1 signaling.

### 3.6 AKT-dependent phosphorylation of ZDHHC5 promotes NOD1 signaling

**Figure 5:**
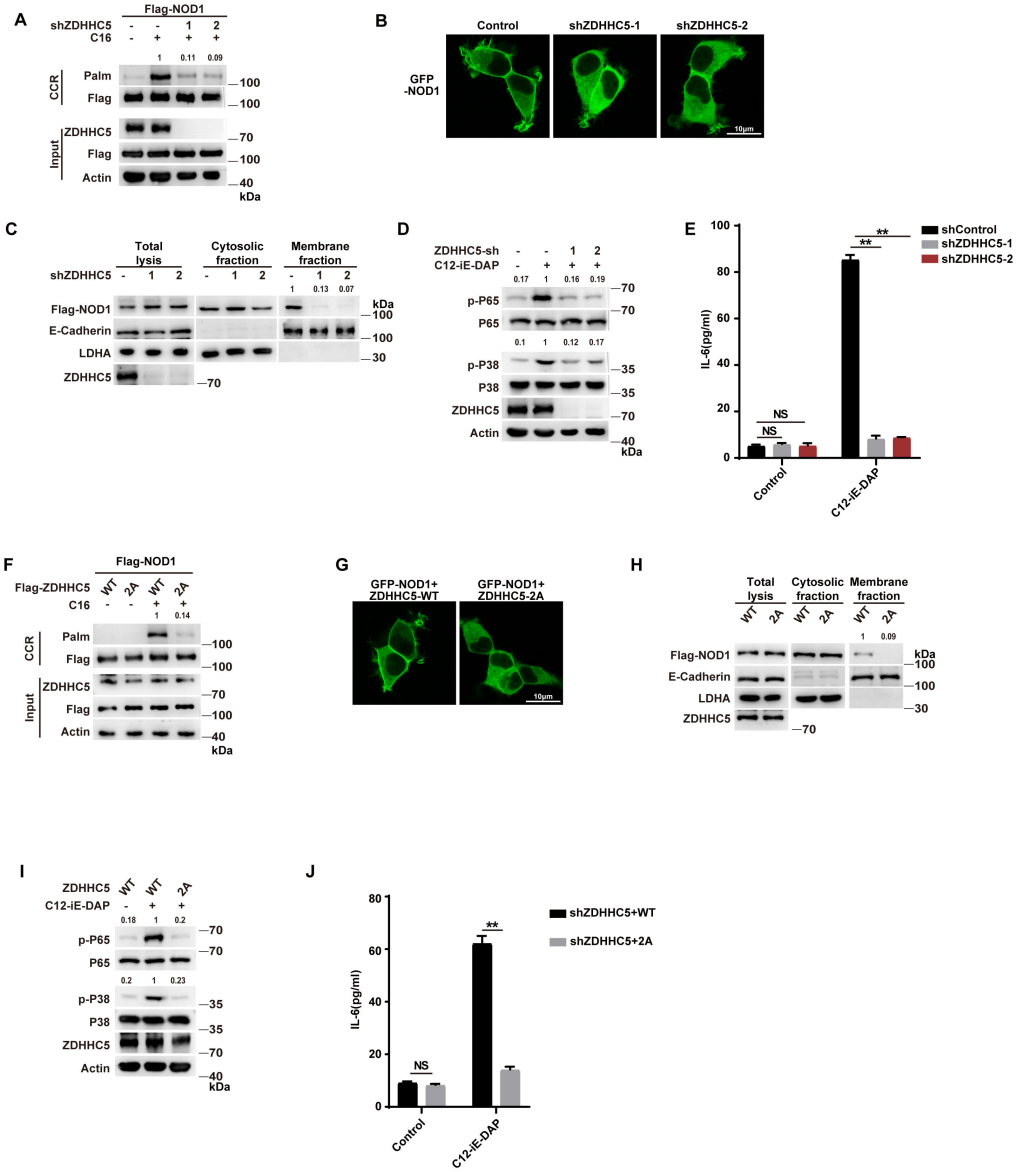
AKT-dependent phosphorylation of ZDHHC5 promotes NOD1 signaling. **(A)** ZDHHC5-knockdown HEK293T cells expressing FLAG-NOD1 were labelled with alk-C16 for 8 h. The NOD1 palmitoylation level was detected using click chemistry reaction. **(B)** Representative fluorescence images showing the localization of GFP-NOD1 in ZDHHC5-knockout HEK293T cells were presented. **(C)** Total, cytosolic, and membrane fractions of ZDHHC5-knockout HEK293T cells expressing FLAG-NOD1 were immunoblotted with indicated antibodies. **(D)** ZDHHC5-knockdown iBMDMs cells were stimulated with C12-iE-DAP (1 μg/ml) for 30 min, p65 and p38 phosphorylation were analyzed by immunoblotting. **(E)** ZDHHC5-knockout iBMDMs cells were stimulated with C12-iE-DAP (5 μg/ml) for 7 h. The IL-6 release in the medium was measured with ELISA. For each experimental group, three replicates were analyzed. **p < 0.01, NS, p > 0.05. mean ± SEM, two-way ANOVA followed by Sidak’s multiple comparisons test. **(F)** ZDHHC5-knockout HEK293T cells reconstituted with ZDHHC5 wild-type (WT) or 2A mutant were transfected to express FLAG-NOD1, then labelled with alk-C16 for 8 h. NOD1 palmitoylation was detected by click chemistry reaction. **(G)** ZDHHC5-knockout HEK293T cells reconstituted with ZDHHC5 wild-type (WT) or 2A mutant. Representative fluorescence images showing the localization of GFP-NOD1 were presented. **(H)** ZDHHC5-knockout HEK293T cells reconstituted with ZDHHC5 wild-type (WT) or 2A mutant were transfected to express FLAG-NOD1. Total, cytosolic, and membrane fractions were immunoblotted with indicated antibodies. **(I)** ZDHHC5-knockdown iBMDMs were reconstituted with ZDHHC5 wild-type (WT) or 2A mutant using lentiviral transduction. The reconstituted iBMDM cells were stimulated with C12-iE-DAP (1 μg/ml) for 30 min, p65 and p38 kinase phosphorylation were analyzed by immunoblotting. **(J)** ZDHHC5-knockdown iBMDMs were reconstituted with ZDHHC5 wild-type (WT) or 2A mutant using lentiviral transduction. The reconstituted iBMDMs cells were stimulated with C12-iE-DAP (5 μg/ml) for 7 h. The IL-6 release in the medium was measured with ELISA. For each experimental group, three supernatant samples were analyzed. **p < 0.01, NS, p > 0.05. mean ± SEM, two-way ANOVA followed by Sidak’s multiple comparisons test.

To establish the functional consequence of ZDHHC5 in NOD1 palmitoylation and downstream signaling, we assessed NOD1 palmitoylation in ZDHHC5-deficient cells. In ZDHHC5-knockdown HEK293T cells expressing FLAG-NOD1, click chemistry revealed a marked reduction in NOD1 palmitoylation compared with control cells (**Figure 5A**). Consistently, fluorescence imaging of GFP-NOD1 in ZDHHC5-knockout HEK293T cells showed diminished enrichment of NOD1 at the membrane (**Figure 5B**; **Supplementary Figures S5A**), which was further confirmed by biochemical fractionation, revealing reduced membrane-associated NOD1 (**Figure 5C**). Accordingly, in ZDHHC5-knockdown iBMDMs, stimulation with the NOD1 agonist C12-iE-DAP substantially attenuated phosphorylation of p65 and p38 compared with control cells (**Figure 5D**). This signaling defect led to diminished effector responses, as reflected by significantly reduced IL-6 secretion in ZDHHC5-knockout iBMDMs after prolonged C12-iE-DAP stimulation. (**Figure 5E**), highlighting the essential role of ZDHHC5 in NOD1-mediated signaling in immune-competent cells.

We reconstituted ZDHHC5-knockout HEK293T cells with either wild-type (WT) ZDHHC5 or a phosphorylation-deficient 2A mutant to assess the necessity of AKT-mediated phosphorylation for these effects. Re-expression of WT ZDHHC5 restored NOD1 palmitoylation, whereas the 2A mutant failed to do so, as detected by click chemistry assay (**Figure 5F**). Concordantly, WT ZDHHC5, but not the 2A variant, rescued the membrane localization of GFP-NOD1, as revealed by both fluorescence microscopy and subcellular fractionation (**Figure 5G**; **Supplementary Figures S5B, H**).

Lentiviral reconstitution of ZDHHC5-knockdown iBMDMs with wild-type ZDHHC5, but not the phosphorylation-deficient 2A mutant, led to recovery of C12-iE-DAP–induced phosphorylation of p65 and p38, as well as IL-6 production (**Figure 5I**; **Supplementary Figures S5C, J**). Collectively, these data demonstrate that AKT-dependent phosphorylation of ZDHHC5 is required to support NOD1 palmitoylation, membrane targeting, and the propagation of downstream innate immune signaling.

## 4 Discussion

Extensive evidence has established that NOD1 signaling is regulated not only by microbial ligands but also by intracellular cues (3, 35). Here, we demonstrate that cellular growth factor and insulin critically regulate NOD1 signaling through the AKT–ZDHHC5 axis. Our data show that AKT activation promotes NOD1 palmitoylation and membrane localization by directly phosphorylating ZDHHC5 at specific serine residues. This modification enhances ZDHHC5 palmitoylation, facilitates its recruitment to the membrane, and enables efficient palmitoylation of NOD1, thereby supporting downstream NF-κB and MAPK activation. In contrast, AKT inhibition or expression of a phosphorylation-deficient ZDHHC5 mutant diminishes NOD1 palmitoylation, impairs membrane targeting, and reduces cytokine production. These results reveal a previously unrecognized AKT–ZDHHC5–NOD1 regulatory axis that connects growth factor–dependent signaling to innate immune activation.

In the canonical NOD1 pathway, membrane recruitment is essential for oligomerization and RIPK2 engagement, both of which are required for downstream signaling (36). Our results demonstrate that AKT activity indirectly regulates NOD1 palmitoylation through phosphorylating ZDHHC5, revealing a post-translational mechanism that links extracellular growth cues with intracellular immune responsiveness. This mechanism is reminiscent of other metabolic regulatory pathways in innate immunity (37, 38), where energy or nutrient signals adjust pattern recognition receptor function to align inflammatory output with cellular metabolic capacity. By linking growth factor signaling to palmitoylation control, the AKT–ZDHHC5 axis ensures that innate immune responses are activated only when metabolic resources are sufficient, thereby preventing inappropriate inflammation under nutrient stress. Moreover, the involvement of two metabolically sensitive enzymes, AKT and ZDHHC5, in NOD1 regulation suggests that additional metabolic enzymes or post-translational modifications may contribute to the fine-tuning of innate immune signaling.

Interestingly, our data indicate that phosphorylation of ZDHHC5 is regulated in a context-dependent manner by distinct metabolic kinases. In our previous study (39), AMPK phosphorylates Ser296 and Ser380 under glucose starvation, inhibiting ZDHHC5-dependent NOD1 palmitoylation and signaling. In contrast, in the present study, AKT phosphorylates Ser345 and Ser380 under growth-supportive conditions, promoting NOD1 palmitoylation and membrane localization. Further localization analyses of individual phospho-Ser mutants revealed that the AKT phosphorylation-deficient mutant S345A displayed markedly reduced plasma membrane localization compared with WT ZDHHC5, whereas the phosphor-mimetic mutant S345D remained predominantly membrane-associated even in the presence of the AKT inhibitor MK2206 (**Supplementary Figure 5E**). These findings identify Ser345 as a major AKT-responsive site regulating ZDHHC5 membrane targeting. In contrast, mutation of Ser380 produced relatively minor effects on membrane localization and downstream NOD1 signaling (**Supplementary Figures 5D, E**), despite partially reducing overall phosphorylation of ZDHHC5 (**Figure 3I**), suggesting that Ser380 functions as a secondary or supportive phosphorylation site rather than the principal functional determinant. The AMPK phosphorylation-deficient mutant S296A retained membrane localization under basal conditions, but still responded to AKT inhibition with reduced membrane localization, indicating that Ser296-mediated AMPK regulation and Ser345-mediated AKT regulation likely represent mechanistically distinct signaling axes. Together, these findings support a model in which ZDHHC5 integrates metabolic cues through site-specific phosphorylation to fine-tune NOD1-mediated signaling under distinct physiological conditions. In this model, Ser345- or Ser296-centered phosphorylation events likely represent the major determinants of NOD1 signaling output, while Ser380 plays a supporting role in modulating phosphorylation context. Consistent with this framework, AMPK and AKT exert opposing effects in metabolic signaling pathways (40, 41), supporting context-dependent regulation of ZDHHC5 activity.

Previous studies have reported aberrant activation of the PI3K–AKT pathway in several autoimmune and inflammatory diseases, where it is associated with excessive innate immune activation and chronic inflammation (25, 42–44). In this context, our findings identify AKT as a critical upstream regulator of ZDHHC5-dependent palmitoylation, which controls NOD1 membrane localization and signaling. These results suggest that dysregulated AKT activity may contribute to pathological NOD1 activation by enhancing ZDHHC5-mediated palmitoylation and signal amplification. Accordingly, pharmacological inhibition of AKT activity may provide a potential strategy to modulate aberrant NOD1 signaling through regulation of the ZDHHC5–NOD1 axis, with potential implications for limiting excessive inflammatory responses in NOD1-driven immune disorders.

Despite these findings, several mechanistic aspects of AKT-ZDHHC5 regulation remain to be clarified. How growth factor-induced AKT signaling spatially and temporally coordinates phosphorylation of ZDHHC5 within distinct cellular compartments warrants further investigation. In addition, the mechanisms by which phosphorylation of ZDHHC5 at Ser345 and Ser380 within its C-terminal cytoplasmic region promotes ZDHHC5 membrane localization remain unclear. These modifications may alter ZDHHC5 conformation or its palmitoylation status, thereby indirectly influencing membrane association. Dissecting how these phosphorylation events alter ZDHHC5 subcellular distribution and enzymatic activity will be an important focus of future studies.

Collectively, our findings integrate growth factor–AKT signaling into the regulatory network of NOD1, revealing that AKT-dependent phosphorylation of ZDHHC5 orchestrates NOD1 palmitoylation, membrane targeting, and downstream inflammatory output. This study expands the repertoire of metabolic and growth factor–dependent mechanisms controlling innate immunity and suggests that targeting the AKT–ZDHHC5–NOD1 axis may provide a therapeutic strategy to modulate inflammation in conditions associated with aberrant NOD1 activation, including metabolic disorders and infectious or inflammatory diseases.

## Data Availability

The mass spectrometry proteomics data have been deposited to the ProteomeXchange Consortium (https://proteomecentral.proteomexchange.org) via the iProX partner repository with the dataset identifier PXD079175. Other data supporting the findings of this study are included in the article and its **Supplementary Materials**.
