# Supplementary material for: AKT-mediated phosphorylation of ZDHHC5 promotes NOD1 palmitoylation and innate immune signaling
Source: Front Immunol. 2026 Jun 9;17:1819627. doi: 10.3389/fimmu.2026.1819627 (PMC13286793; doi:10.3389/fimmu.2026.1819627)
Supplement: Supplementary file 1 [file DataSheet1.pdf]

FIG S1

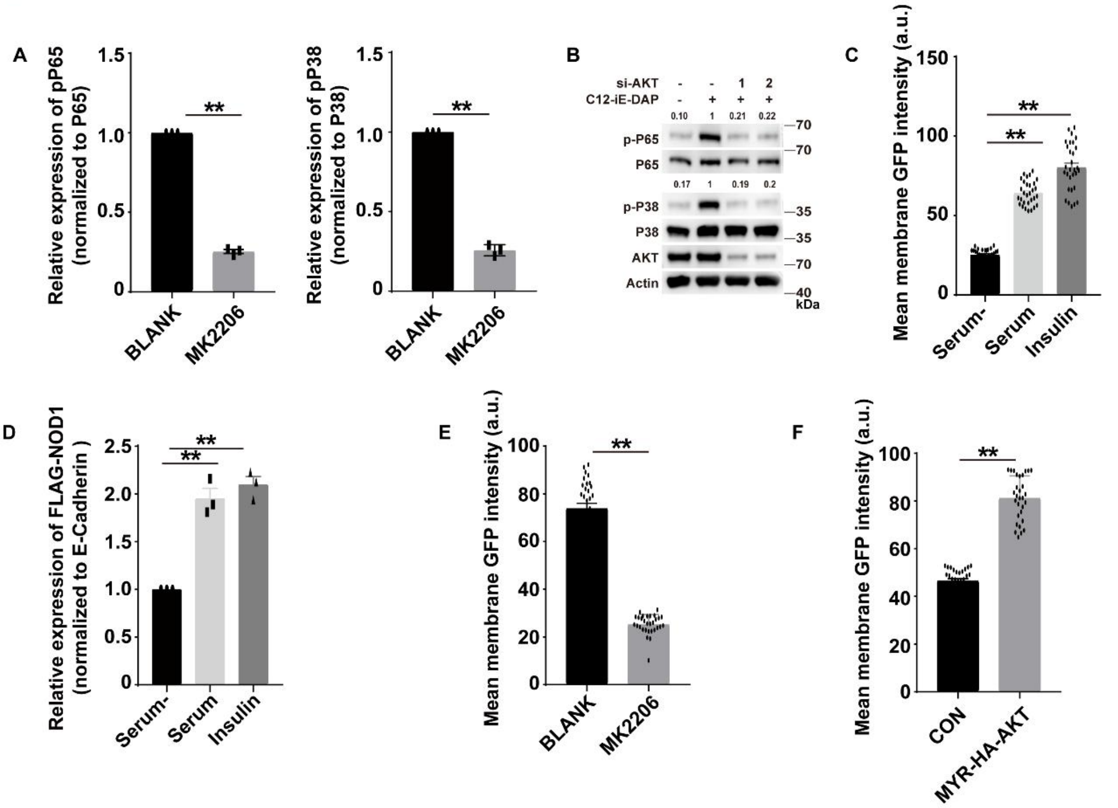

A
Relative expression of pP65 (normalized to P65)
BLANK
MK2206
**
Relative expression of pP38 (normalized to P38)
B
si-AKT
C12-iE-DAP
p-P65
P65
p-P38
P38
AKT
Actin
kDa
C
Mean membrane GFP intensity (a.u.)
Serum-
Serum
Insulin
D
Relative expression of FLAG-NOD1 (normalized to E-Cadherin)
E
F
CON
MYR-HA-AKT

Fig. S1. AKT signaling regulates NOD1-mediated inflammatory signaling and membrane localization.

(**A**) Quantification of p-P65 and p-P38 levels in iBMDMs under the indicated treatment conditions. p-P65 and p-P38 levels were normalized to total P65 and P38 and expressed as fold change relative to control.

(**B**) Mouse iBMDMs cells were transfected with two independent siRNAs targeting AKT1, followed by stimulation with C12-iE-DAP (1 μg/ml) for 30 min. p65 and p38 phosphorylation were analyzed by immunoblotting.

(**C**) Quantification of plasma membrane–associated GFP-NOD1 fluorescence intensity in HEK293T cells under the indicated treatment conditions. Individual cells were manually outlined, and a membrane-associated region of interest (ROI) was generated by expanding the selection by 3 pixels. Mean fluorescence intensity within the membrane ROI was quantified on a per-cell basis using ImageJ. The ROI width was kept constant across all conditions. At least 30 cells per condition were analyzed in a blinded manner.

(**D**) Quantification of membrane-associated NOD1 levels normalized to E-cadherin and expressed relative to control under the indicated treatment conditions.

(**E**) Quantification of plasma membrane–associated GFP-NOD1 fluorescence intensity in HEK293T cells under the indicated treatment conditions.

(**F**) Quantification of plasma membrane–associated GFP-NOD1 fluorescence intensity in HEK293T cells with or without MYR-HA-AKT overexpression.

Data are presented as mean ± SEM from three independent experiments ($n = 3$). Statistical analysis was performed using unpaired two-tailed Student’s t-test (for two-group comparisons) or one-way ANOVA followed by Dunnett’s multiple comparisons test (for multiple-group comparisons). $^{**}p < 0.01$; ns, not significant.

FIG S2

A

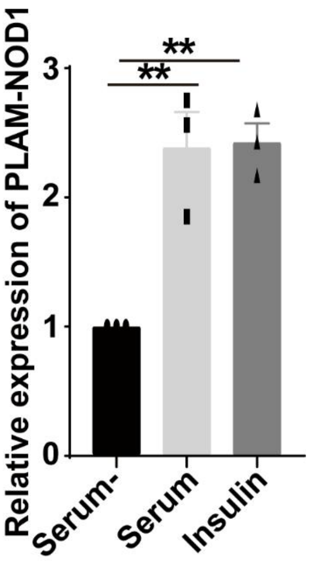

Fig. S2. AKT activation enhances NOD1 palmitoylation

(**A**) Quantification of palmitoylation of NOD1 levels normalized to FLAG-NOD1 and expressed relative to control under different treatment conditions as indicated.

Data are presented as mean ± SEM from three independent experiments ($n = 3$). Statistical analysis was performed using one-way ANOVA followed by Dunnett's multiple comparisons test, as appropriate. **$p < 0.01$; ns, not significant.

FIG S3

A

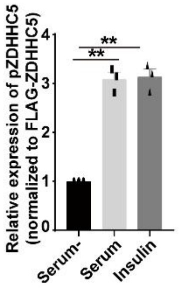

C

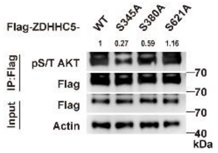

B

Site	Phospho (STY) Probabilities
S345	THLGLAT(0.001)NEDS(0.006)S(0.007)LLAKDS(0.985)PPT(0.859)PT(0.134)MY(0.009)K
S380	GDS(1)LKEPTSIAESSR
S621	GVGS(1)PEPGPTAPYLGR

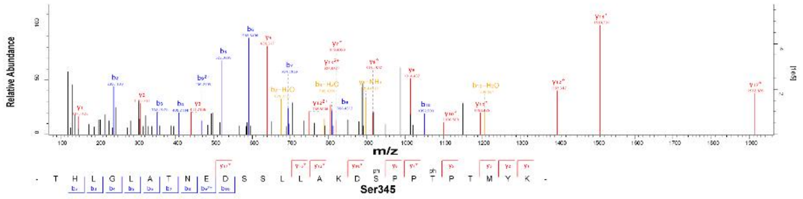

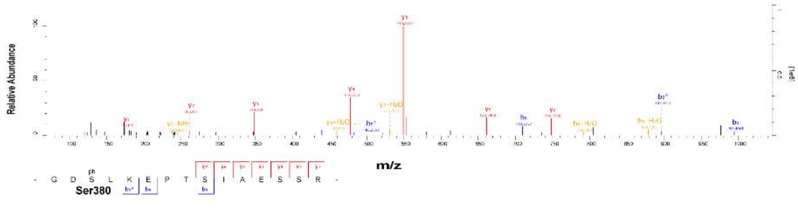

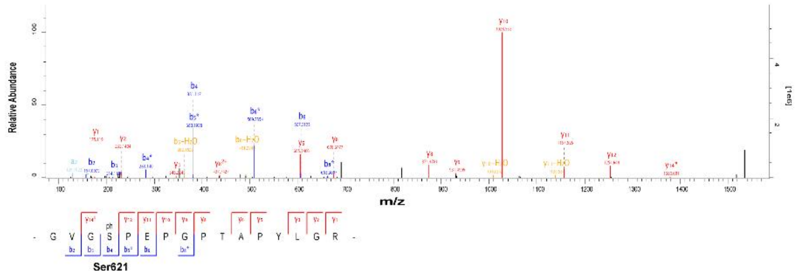

Fig. S3. AKT phosphorylates ZDHHC5

(**A**) Quantification of ZDHHC5 phosphorylation levels normalized to FLAG-ZDHHC5 and expressed relative to control under the indicated treatment conditions.

(**B**) Identification of phosphorylation sites on ZDHHC5 by mass spectrometry. Top panel, phosphorylation (STY) site probabilities of indicated sites with localization probability $\geq$ 0.98 highlighted as red. In MaxQuant, phosphorylation (STY) site probability reflects site assignment reliability rather than peptide identification confidence or phosphorylation stoichiometry. Phosphosites with localization probability $\geq$ 0.75 are commonly regarded as high-confidence (Class I) sites. Bottom panel, the MS/MS spectra for indicated phosphosites with well-resolved b- and y-ions.

(**C**) HEK293T cells were transfected with indicated plasmids, and immunoprecipitation was performed using FLAG M2 beads. The immunoprecipitates were then analyzed by Western blotting with a Phospho-AKT Substrate motif antibody.

Data are presented as mean ± SEM from three independent experiments (n = 3). Statistical analysis was performed using one-way ANOVA followed by Dunnett's multiple comparisons test, as appropriate. **$p < 0.01$; ns, not significant.

FIG S4

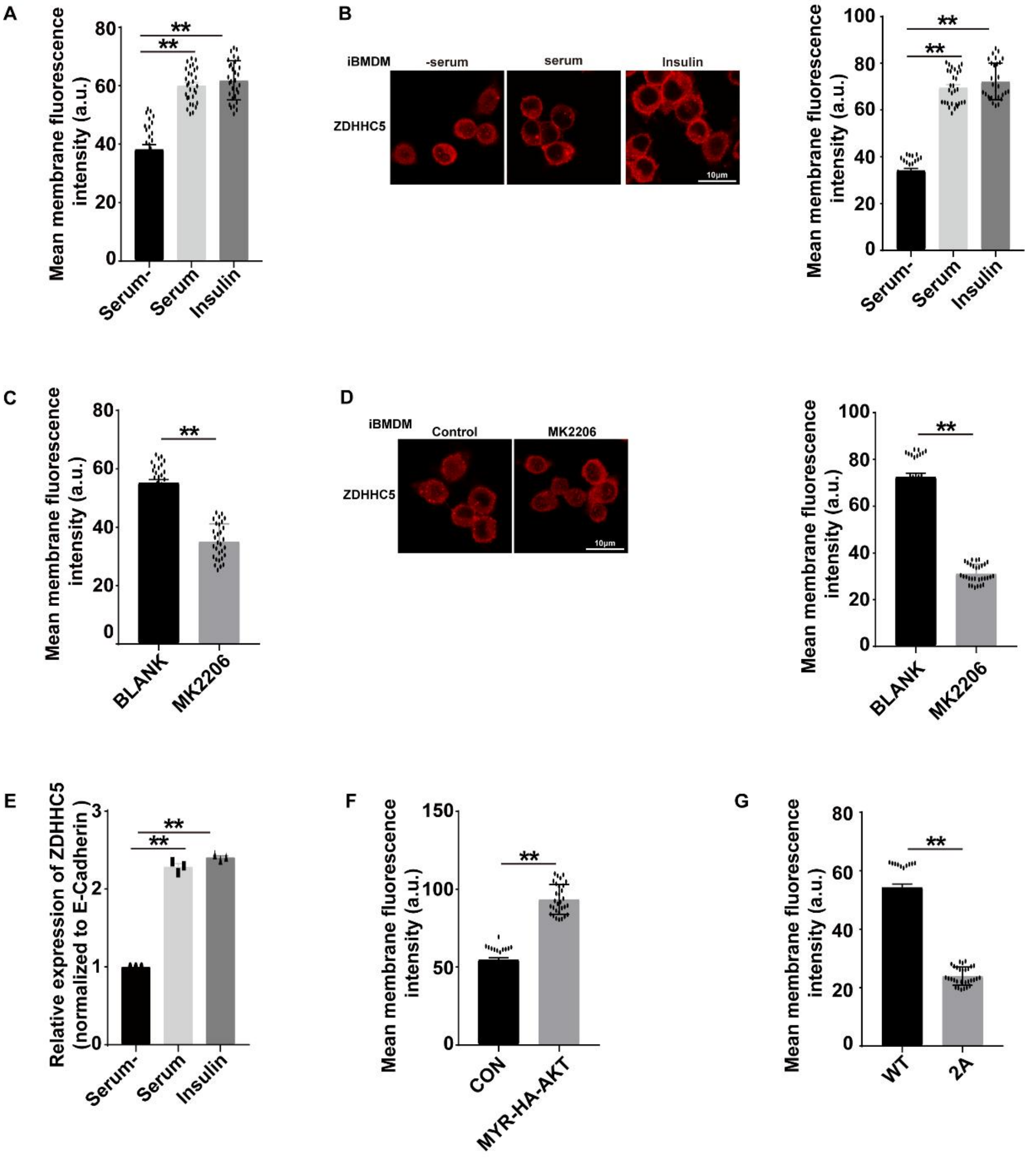

A
Mean membrane fluorescence intensity (a.u.)
80
60
40
20
0
**
**
Serum-
Serum
Insulin
B
iBMDM
-serum
serum
Insulin
ZDHHC5
10μm
Mean membrane fluorescence intensity (a.u.)
100
80
60
40
20
0
**
**
Serum-
Serum
Insulin
C
Mean membrane fluorescence intensity (a.u.)
80
60
40
20
0
**
BLANK
MK2206
D
iBMDM
Control
MK2206
ZDHHC5
10μm
Mean membrane fluorescence intensity (a.u.)
100
80
60
40
20
0
**
BLANK
MK2206
E
Relative expression of ZDHHC5 (normalized to E-Cadherin)
3
2
1
0
**
**
Serum-
Serum
Insulin
F
Mean membrane fluorescence intensity (a.u.)
150
100
50
0
**
CON
MYR-HA-AKT
G
Mean membrane fluorescence intensity (a.u.)
80
60
40
20
0
**
WT
2A

Fig. S4. AKT-dependent phosphorylation regulates plasma membrane localization of ZDHHC5.

(**A**) Quantification of plasma membrane–associated endogenous ZDHHC5 fluorescence intensity in HEK293T cells under the indicated treatment conditions.

(**B**) iBMDM cells were subjected to serum starvation, serum refeeding, or insulin stimulation. Representative fluorescence images (left) and quantification (right) of plasma membrane–associated endogenous ZDHHC5 are shown.

(**C**) Quantification of plasma membrane–associated endogenous ZDHHC5 fluorescence intensity in HEK293T cells under the indicated treatment conditions.

(**D**) iBMDM cells were treated with the AKT inhibitor MK2206 (2 μM) under serum-free conditions. Representative fluorescence images (left) and quantification (right) of plasma membrane–associated endogenous ZDHHC5 are shown.

(**E**) Quantification of membrane-associated ZDHHC5 levels normalized to E-cadherin and expressed relative to control under the indicated treatment conditions.

(**F**) Quantification of plasma membrane–associated endogenous ZDHHC5 fluorescence intensity in HEK293T cells with or without MYR-HA-AKT overexpression.

(**G**) Quantification of plasma membrane–associated FLAG-ZDHHC5 (WT and 2A) fluorescence intensity in HEK293T cells.

Data are presented as mean ± SEM from three independent experiments (n = 3). Statistical analysis was performed using unpaired two-tailed Student's t-test (for two-group comparisons) or one-way ANOVA followed by Dunnett's multiple comparisons test (for multiple-group comparisons). $^{**}p < 0.01$; ns, not significant.

FIG S5

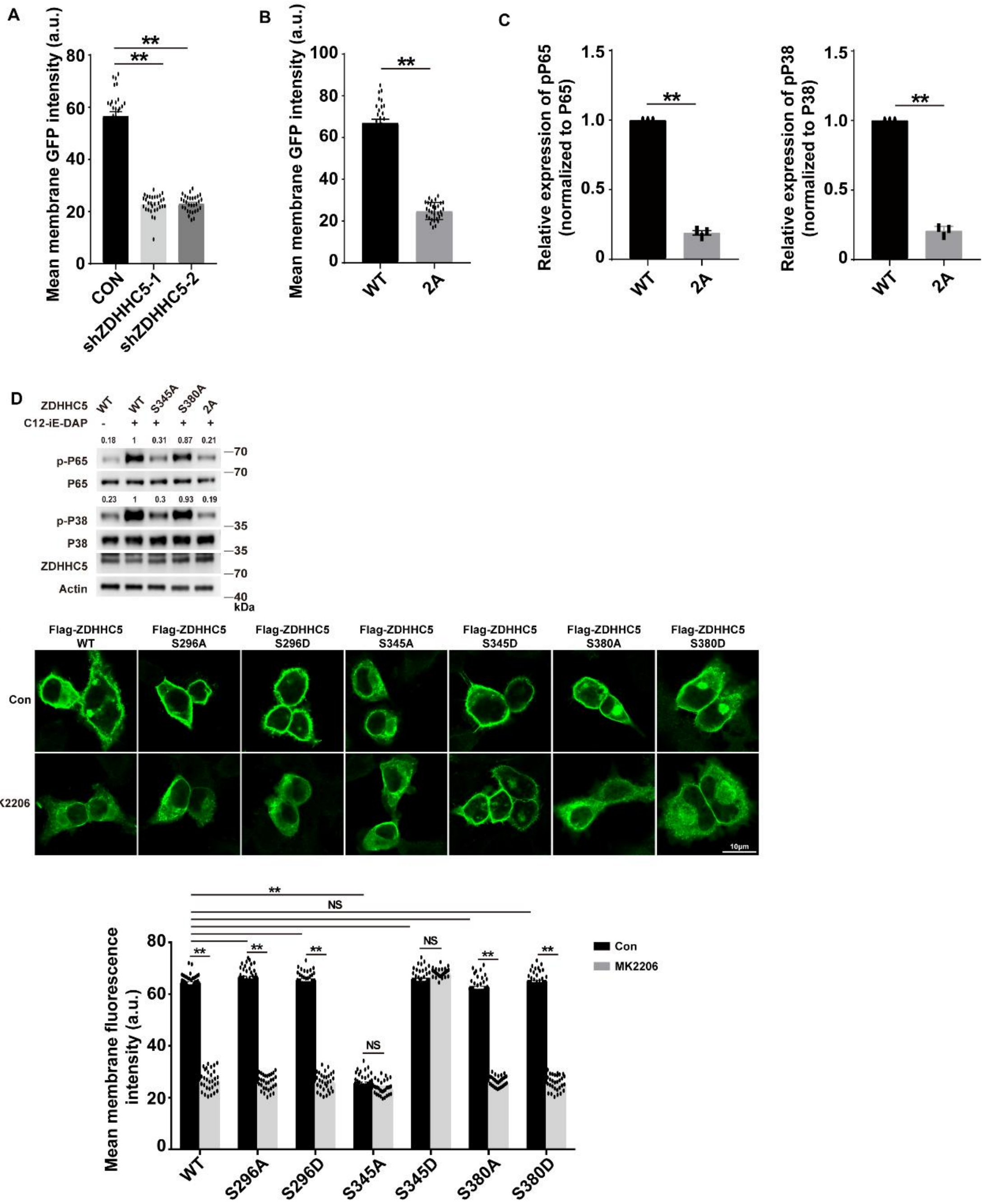
A
Mean membrane GFP intensity (a.u.)
CON
shZDHHC5-1
shZDHHC5-2
B
Mean membrane GFP intensity (a.u.)
WT
2A
C
Relative expression of pP65 (normalized to P65)
Relative expression of pP38 (normalized to P38)
D
ZDHHC5
WT
S345A
S380A
2A
C12-iE-DAP
p-P65
P65
p-P38
P38
Actin
kDa
E
Flag-ZDHHC5 WT
Flag-ZDHHC5 S296A
Flag-ZDHHC5 S296D
Flag-ZDHHC5 S345A
Flag-ZDHHC5 S345D
Flag-ZDHHC5 S380A
Flag-ZDHHC5 S380D
Con
MK2206
10μm
NS
Mean membrane fluorescence intensity (a.u.)
S345D
S380D

Fig. S5. AKT-dependent phosphorylation of ZDHHC5 regulates NOD1 signaling

(**A**) Quantification of plasma membrane–associated GFP-NOD1 fluorescence intensity in HEK293T cells under the indicated gene knockdown.

(**B**) Quantification of plasma membrane–associated GFP-NOD1 fluorescence intensity in HEK293T cells following ZDHHC5 knockdown and reconstitution with WT or 2A mutant ZDHHC5.

(**C**) Quantification of p-P65/p-P38 levels in ZDHHC5-knockdown iBMDM cells following reconstitution with WT or 2A mutant ZDHHC5. Protein expression was normalized to P65/P38 and shown as fold change relative to control.

(**D**) ZDHHC5-knockdown iBMDMs were reconstituted with lentiviral expression of ZDHHC5 WT or the indicated mutants. The reconstituted iBMDM cells were stimulated with C12-iE-DAP (1 μg/ml) for 30 min, p65 and p38 kinase phosphorylation were analyzed by immunoblotting.

(**E**) HEK293T cells were transfected with indicated plasmids and subsequently treated with the AKT inhibitor MK2206 (2 μM) under serum-free conditions. Representative fluorescence images (up) and quantification (down) of plasma membrane–associated FLAG-ZDHHC5 are shown. Statistical analysis was performed using two-way ANOVA followed by Sidak's multiple comparisons test (for comparisons involving two independent variables). $**p < 0.01$; ns, not significant.

Data are presented as mean ± SEM from three independent experiments ($n = 3$). Statistical analysis was performed using unpaired two-tailed Student's t-test (for two-group comparisons), one-way ANOVA followed by Dunnett's multiple comparisons test (for multiple-group comparisons), or two-way ANOVA followed by Sidak's multiple comparisons test (for comparisons involving two independent variables). $**p < 0.01$; ns, not significant.
