# Supplementary material for: AKT-mediated phosphorylation of ZDHHC5 promotes NOD1 palmitoylation and innate immune signaling
Source: Front Immunol. 2026 Jun 9;17:1819627. doi: 10.3389/fimmu.2026.1819627 (PMC13286793; doi:10.3389/fimmu.2026.1819627)

Representative western blot images from three independent experiments are shown, and quantitative statistical analyses were performed for all western blot and immunofluorescence data.
Band intensities in Western blots were quantified using ImageJ software. Protein levels were normalized to the corresponding loading control (e.g., E-cadherin).
Quantification was performed from at least three independent experiments. Quantification of immunofluorescence signals was performed using ImageJ.
Individual cells were manually outlined, and regions of interest (ROIs) were defined consistently across all samples.
Mean fluorescence intensity was measured for each cell, and at least 30 cells per condition were analyzed. Data were collected from three independent experiments

FIG 1

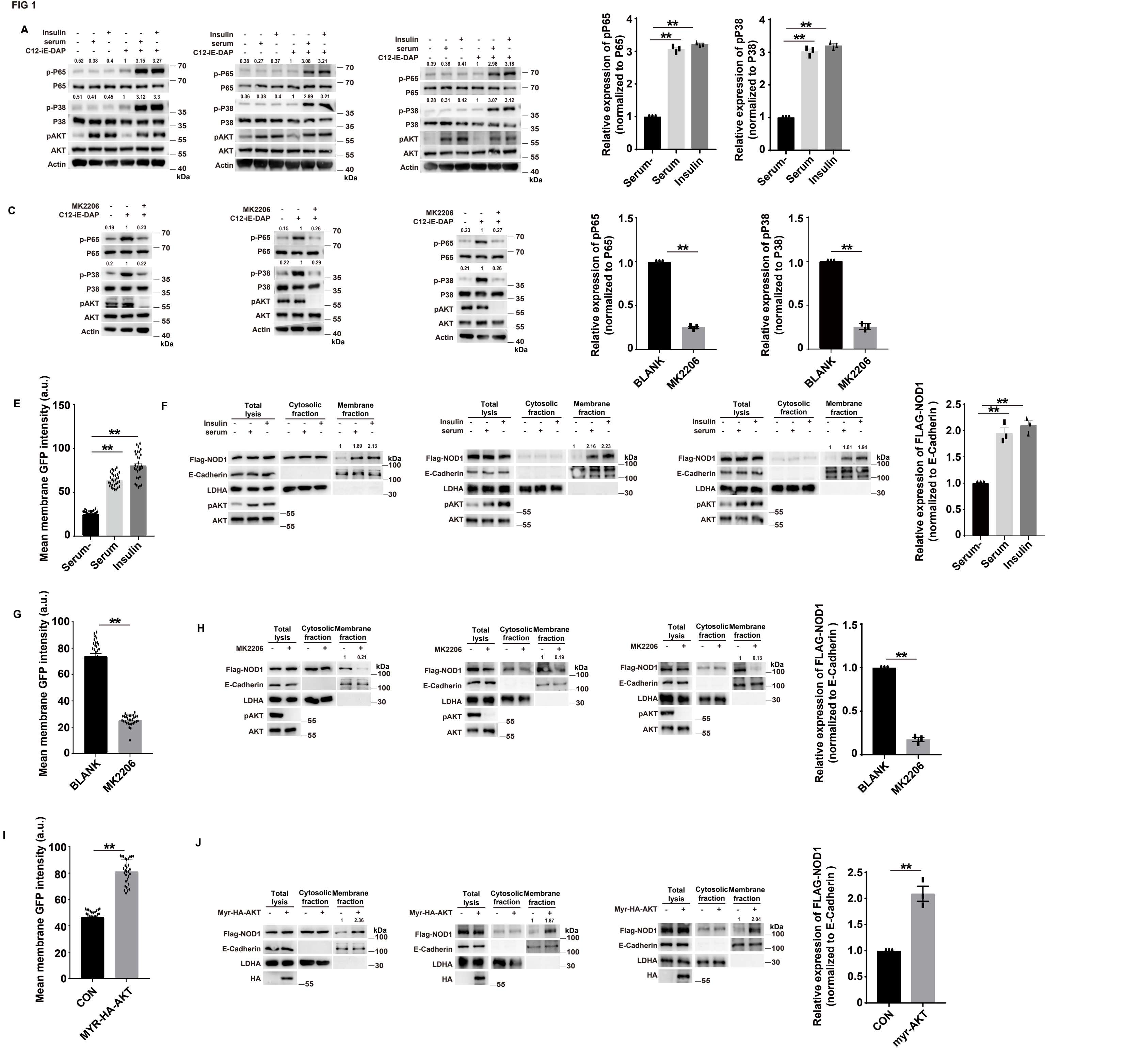

FIG 2

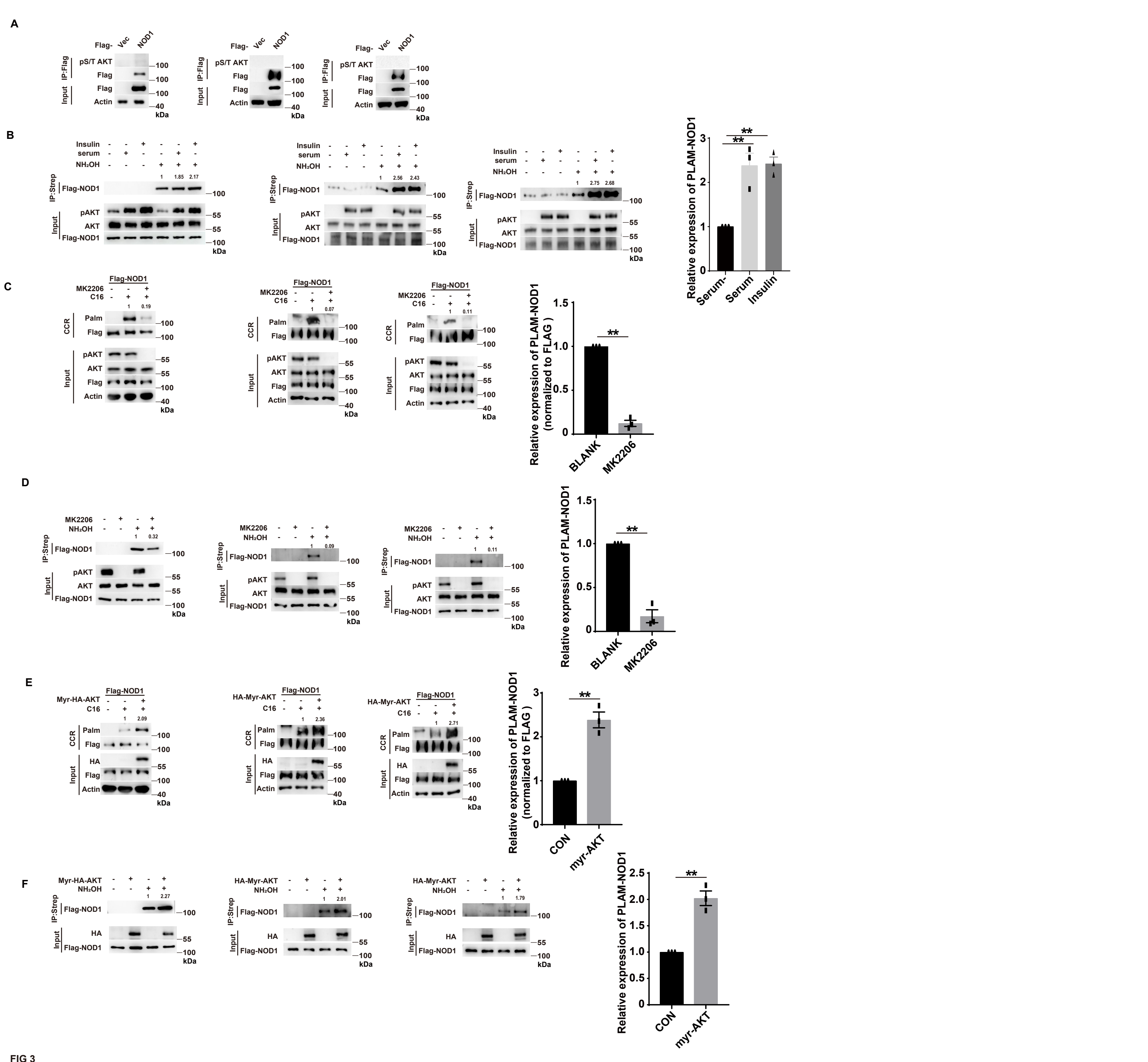

FIG 3

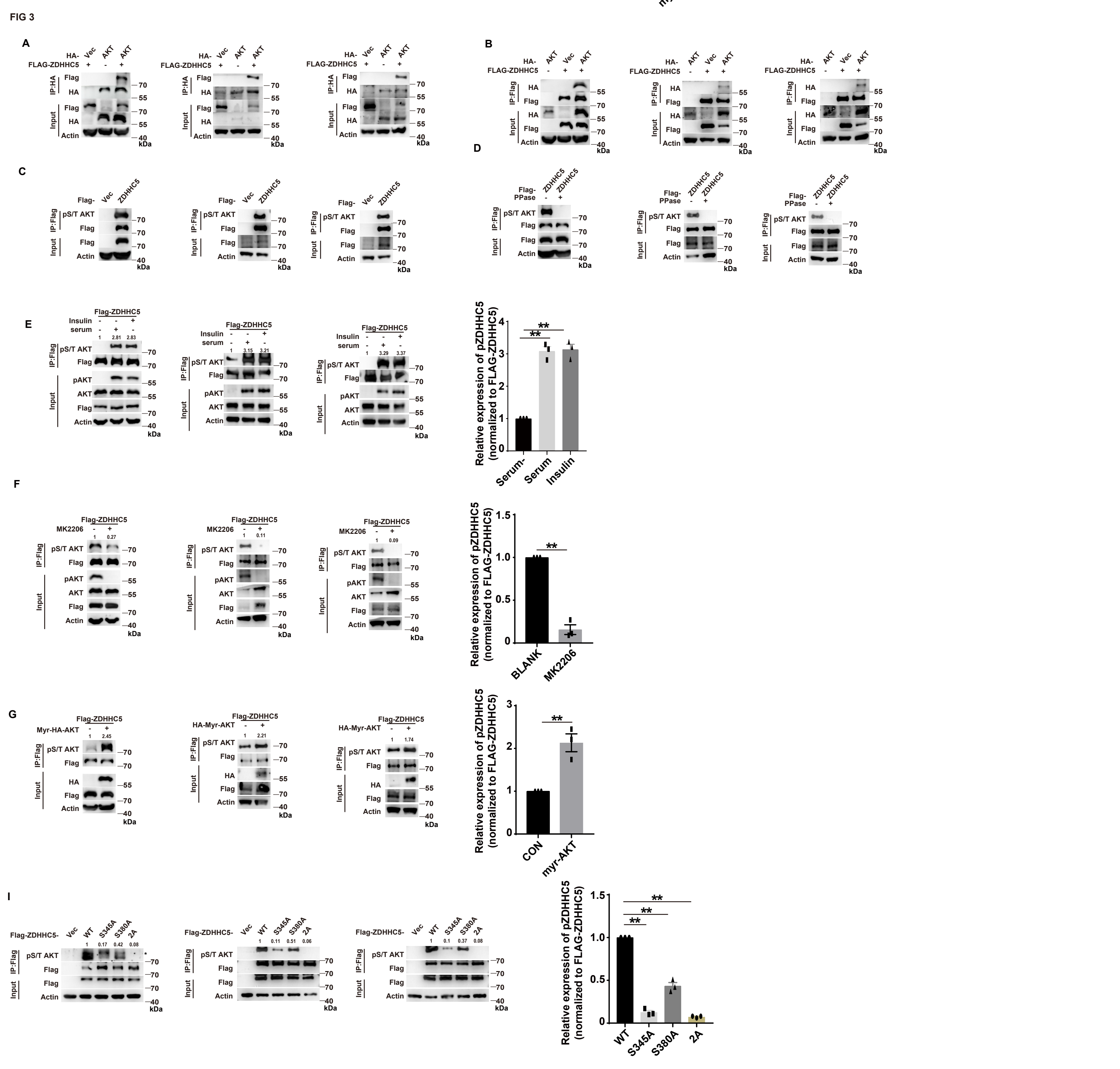

FIG 4

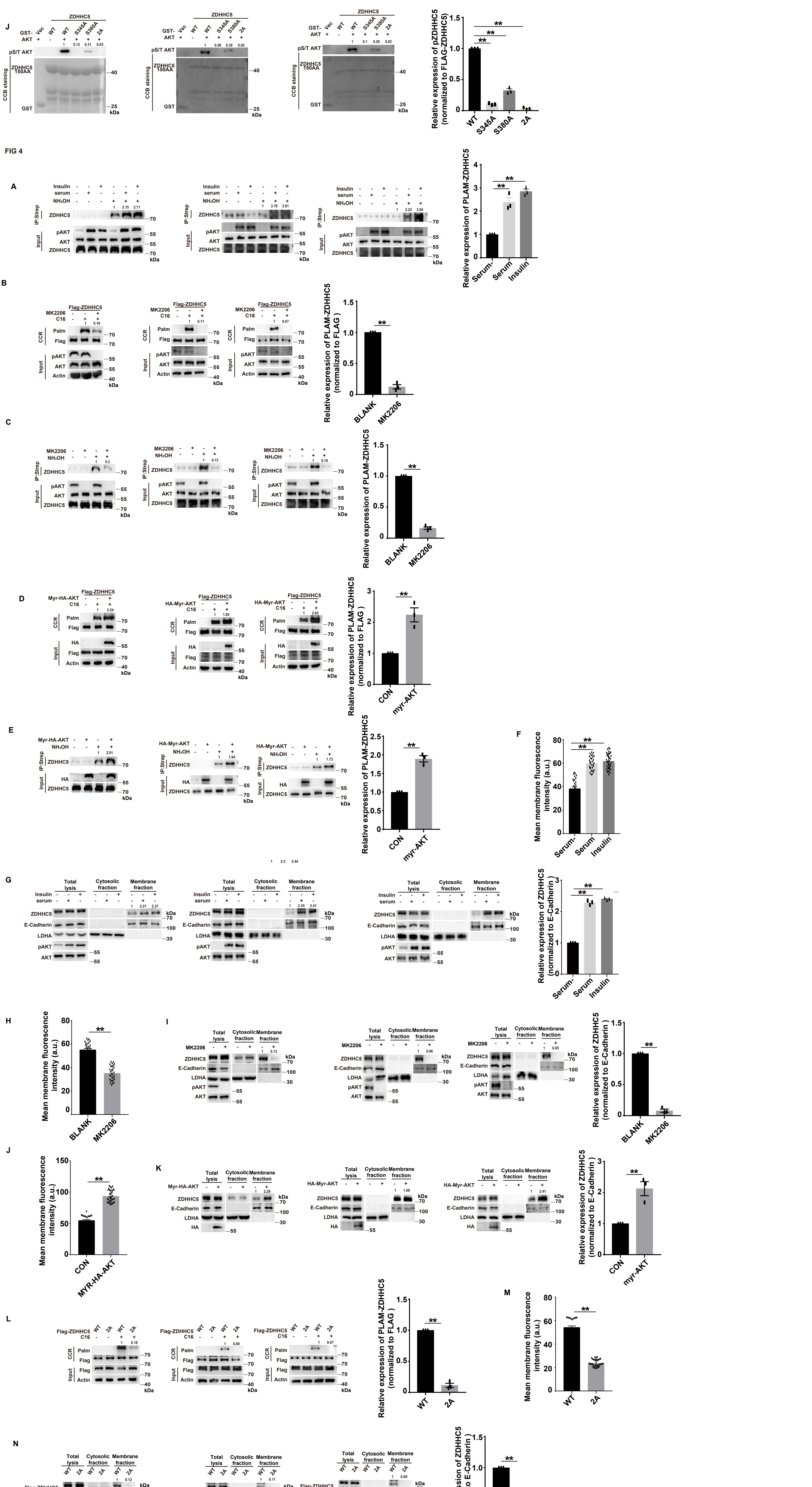

FIG 5

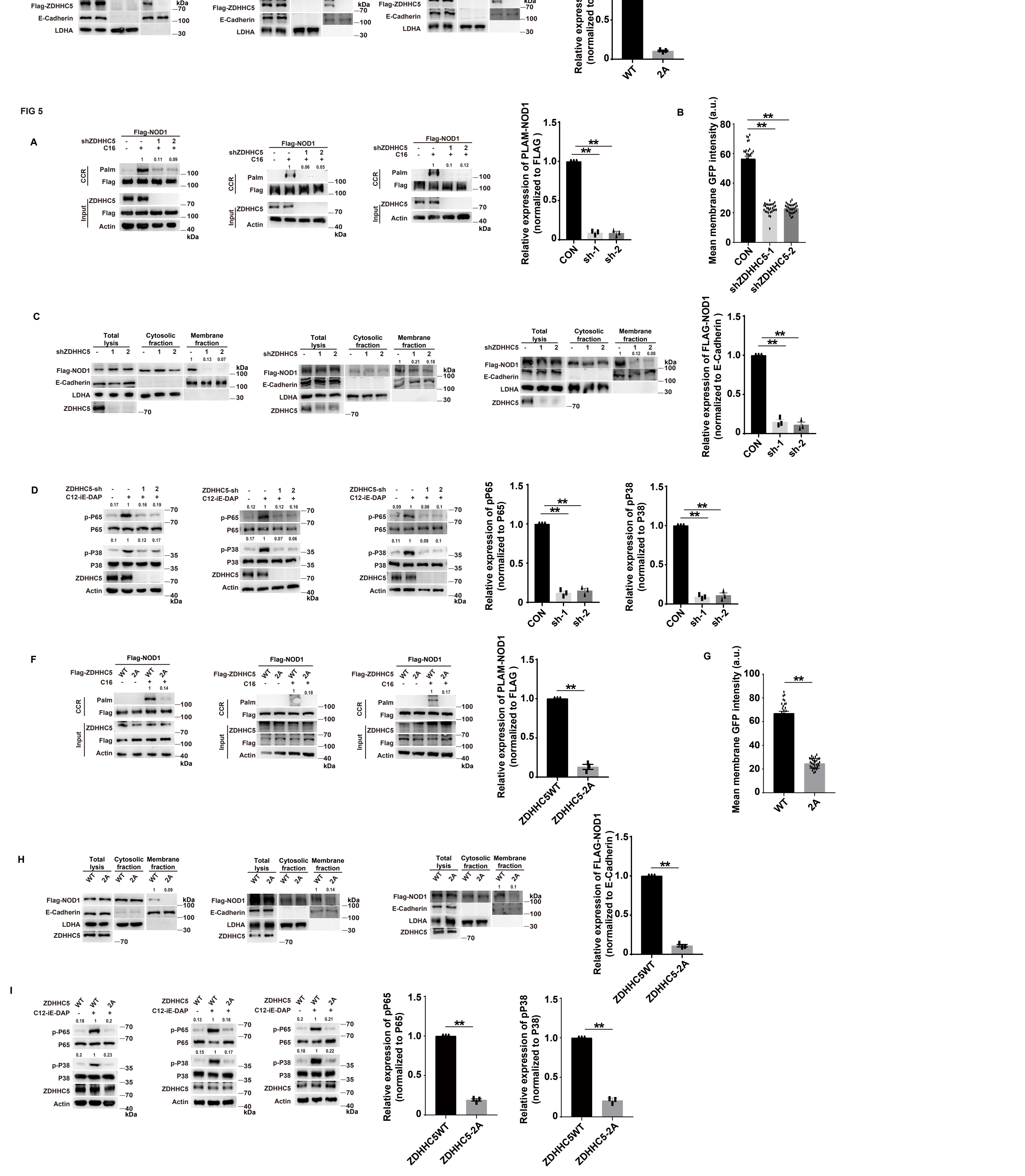

Supplement: Supplementary file 2 [file DataSheet2.pdf]
